# Calprotectin blockade inhibits long-term vascular pathology following peritoneal dialysis-associated bacterial infection

**DOI:** 10.3389/fcimb.2023.1285193

**Published:** 2023-11-29

**Authors:** Esra Cetin, Morgane Mazzarino, Guadalupe T. González-Mateo, Valeria Kopytina, Soma Meran, Donald Fraser, Manuel López-Cabrera, Mario O. Labéta, Anne-Catherine Raby

**Affiliations:** ^1^ Wales Kidney Research Unit, Division of Infection & Immunity, School of Medicine, Cardiff University, Cardiff, United Kingdom; ^2^ Tissue and Organ Homeostasis Program, Centro de Biología Molecular Severo Ochoa – Consejo Superior de Investigaciones Científicas – Universidad Autónoma de Madrid (CBMSO-CSIC-UAM), Madrid, Spain; ^3^ Premium Research, S.L., Guadalajara, Spain

**Keywords:** infection, vascular inflammation, damage associated moeleular patterns, peritoneal dialysis, anti-inflammatory intervention strategies

## Abstract

Bacterial infections and the concurrent inflammation have been associated with increased long-term cardiovascular (CV) risk. In patients receiving peritoneal dialysis (PD), bacterial peritonitis is a common occurrence, and each episode further increases late CV mortality risk. However, the underlying mechanism(s) remains to be elucidated before safe and efficient anti-inflammatory interventions can be developed. Damage-Associated Molecular Patterns (DAMPs) have been shown to contribute to the acute inflammatory response to infections, but a potential role for DAMPs in mediating long-term vascular inflammation and CV risk following infection resolution in PD, has not been investigated. We found that bacterial peritonitis in mice that resolved within 24h led to CV disease-promoting systemic and vascular immune-mediated inflammatory responses that were maintained up to 28 days. These included higher blood proportions of inflammatory leukocytes displaying increased adhesion molecule expression, higher plasma cytokines levels, and increased aortic inflammatory and atherosclerosis-associated gene expression. These effects were also observed in infected nephropathic mice and amplified in mice routinely exposed to PD fluids. A peritonitis episode resulted in elevated plasma levels of the DAMP Calprotectin, both in PD patients and mice, here the increase was maintained up to 28 days. *In vitro*, the ability of culture supernatants from infected cells to promote key inflammatory and atherosclerosis-associated cellular responses, such as monocyte chemotaxis, and foam cell formation, was Calprotectin-dependent. *In vivo*, Calprotectin blockade robustly inhibited the short and long-term peripheral and vascular consequences of peritonitis, thereby demonstrating that targeting of the DAMP Calprotectin is a promising therapeutic strategy to reduce the long-lasting vascular inflammatory aftermath of an infection, notably PD-associated peritonitis, ultimately lowering CV risk.

## Introduction

Bacterial infections have been associated with increased cardiovascular (CV) risk in the short (Clayton et al., 2007; Chen et al., 2012; Dalager-Pedersen et al., 2014) and long (Chen et al., 2012; Bergh et al., 2017) term. While serious infections carry the biggest risk (Jafarzadeh et al., 2016), less severe local infections, such as periodontal infections, were also found to increase long-term CV risk (Sanz et al., 2020; Zardawi et al., 2020), notably via the promotion of atherosclerosis (Beukers et al., 2017).

Chronic Kidney Disease (CKD) at all stages is associated with significantly increased cardiovascular (CV) risk (Gansevoort et al., 2013; Liu et al., 2014), with patients on dialysis showing a 20-fold increase in CV mortality (Gansevoort et al., 2013). In CKD patients receiving peritoneal dialysis (PD), bacterial peritonitis is a common occurrence, and increasing evidence suggests an additional promoting long-term effect of prior peritonitis episodes on late CV mortality (Ye et al., 2017; Pecoits-Filho et al., 2018; Cheikh Hassan et al., 2022). In a 3-year follow-up period, peritonitis was associated with a 90% increased risk of cardiovascular mortality (Ye et al., 2017), and increasing number of peritonitis episodes were found to further increase CV mortality risk in PD patients (Pecoits-Filho et al., 2018; Cheikh Hassan et al., 2022).

Increasing evidence suggests that infection-induced inflammation, rather than the pathogen itself, promotes CV pathologies (Bergh et al., 2017; Pecoits-Filho et al., 2018). Therefore, anti-inflammatory therapies administered during or after infections such as PD-associated peritonitis may be valuable adjuncts to standard anti-microbial therapies to reduce long-term CV risk. Development of novel anti-inflammatory strategies in PD will, however, require elucidation of the mechanisms by which acute infection-induced inflammatory responses transition to lasting low grade vascular inflammation and increased CV risk in these patients.

Endogenous components released and/or generated as a result of cellular stress and tissue damage, termed Damage-Associated Molecular Patterns (DAMPs), which interact with pro-inflammatory Pattern-Recognition Receptors (PRRs), are critical to the development of chronic inflammatory pathologies, including CV pathologies, such as atherosclerosis (Tobias and Curtiss, 2007; Feldman et al., 2015; Roshan et al., 2016; Roh and Sohn, 2018). In addition to their role in mediating sterile inflammation, DAMPs have also been shown to significantly contribute to the inflammatory response to severe bacterial infections. Notably, elevated plasma levels of the host DAMPs mitochondrial DNA (Yamanouchi et al., 2013) and Calprotectin (S100A8/S100A9) (Wirtz et al., 2020; Parke et al., 2023) in sepsis have been found to correlate with disease severity. Furthermore, deficiency in or blockade of S100A9, reduced organ damage during sepsis (Ding et al., 2021; Wu et al., 2023; Zhang et al., 2023). However, the potential role of specific DAMPs in mediating lasting inflammation and increased CV risk long past infection resolution, including following peritonitis in PD, remains to be evaluated.

The present study revealed that a single peritoneal bacterial infection episode in mice leads to systemic and vascular inflammatory changes that are maintained 28 days past infection resolution and can promote vascular inflammation and atherosclerosis development by inducing i) higher blood proportion of inflammatory innate immune leukocytes, ii) increased leukocyte expression of adhesion molecules, ii) higher plasma pro-inflammatory cytokine levels, and iv) increased aortic inflammatory and atherosclerosis-associated gene expression. Importantly in the context of PD-associated peritonitis, these observations were reproduced in nephropathic mice and aggravated upon repeated daily exposure to PD fluids. A peritonitis episode resulted in a strong elevation in plasma levels of the DAMP Calprotectin, both in PD patients and mice, which in the latter remained elevated for at least 28 days. *In vitro*, the ability of culture supernatants from infected cells to promote typical key vascular inflammatory and pro-atherosclerotic responses, such as monocyte chemotaxis, and foam cell formation, was demonstrated to be Calprotectin-dependent. *In vivo*, Calprotectin blockade robustly inhibited the short and long-term systemic and vascular inflammatory consequences of peritonitis in mice, without affecting bacterial clearance.

Thus, our findings demonstrate that specific targeting of the DAMP Calprotectin is a promising therapeutic strategy to reduce the long-lasting peripheral and vascular inflammatory aftermath of an infection, notably peritonitis in PD, ultimately lowering CV risk.

## Methods

### Animal work

All experiments were carried out under a Home Office project licence. 7- to 9- week old female wild-type mice (C57BL/6, n=5/group/time point, unless indicated otherwise) were i.p. injected with live *S. epidermidis* at a dose around 5x10^8^ cfu/mouse, as previously defined by our laboratory (Raby et al., 2009; Raby et al., 2017). Live inoculum was prepared based on previously established standard curves correlating the turbidity (OD_600_) of the bacterial suspension with the number of bacterial colonies after growth on Petri dishes. Therefore, while mice were always injected with the same OD inoculum, the actual number of colonies injected was only confirmed 1-2 days later following culture of the inoculum and may vary slightly between experiments. The actual number of bacteria injected for each experiment (Time course, 6.8x10^8^ cfu/mouse; AAN mice, 7.8x10^8^ cfu/mouse; PDF-exposed mice, 3.85x10^8^ cfu/mouse; Paquinimod treatment, 8.75x10^8^ cfu/mouse).

For Calprotectin blockade experiments, PBS or live *S. epidermidis* i.p. injections were performed in the presence or absence of Paquinimod (1 mg/kg) and Paquinimod was repeated weekly until cull date, for a total of 3 additional injections. Mice were sacrificed at the indicated time points and blood (cardiac puncture) and peritoneal lavages (in 2ml PBS) were collected. The heart was flushed with PBS (10 ml) prior to aorta isolation. The thoracic portion of the aorta was immediately snap-frozen prior to RNA extraction. Aliquots of blood (30µl) and lavage (300µl) from each mouse were set aside for cell surface staining and the rest of the samples were centrifuged at 450 x g for 15 mins at 4°C to obtain plasma and cell-free lavage. Samples were stored at -80˚C until analysis by ELISA or for creatinine levels (Cardiff and Vale UHB, Medical Biochemistry services). Total RNA was extracted from blood using the QIAamp RNA Blood Mini Kit (Qiagen).

Chronic Aristolochic Acid-induced Nephropathy (AAN) was induced as previously described (Lu et al., 2021). 7- to 9- week old female wild-type mice (C57BL/6, n=5/group/time point) were i.p. injected with PBS (500 μl) or Aristolochic Acid (AA, Sigma, 2.5 mg/kg). Injections were repeated every 3 days for a total of 4 times. 28 days after the first AA injection, mice from each group were injected i.p. with live *S. epidermidis* or PBS (Day 0) and culled at Day 1 or Day 28. Bacterial injection, tissue collection and sample processing were as described above. In addition, the right kidney was isolated and halved lengthwise, transferred to a histology cassette and fixed for at least 24h (4°C) in 10% Neutral-Buffered Formalin (NBF) before being transferred to 70% Ethanol (4°C) until embedding, slicing (8µm) and Masson’s trichrome staining (Bioimaging Hub, Cardiff University).

PD fluid (PDF) exposure experiments were performed under a project license granted by the Spanish Consejo Superior de Investigaciones Científicas. 7- to 9-week-old female wild-type mice (C57BL/6, n=6/group/time point) underwent peritoneal catheter (Access technologies, West Midlands, UK) implantation surgery, as previously reported (Gonzalez-Mateo et al., 2009; Raby et al., 2018a). Following 7 days of recovery, mice were instilled once daily with 2ml PBS (n=24) or 2ml PDF (n=24, Fresenius standard 4.25% glucose solution) for 14 days. Mice were then i.p. injected with *S. epidermidis* or PBS (Day 0) and PBS and PDF instillation were carried out daily until cull date (Day 1 or Day 28). Blood, peritoneal lavages and aortas were collected and processed as described above.

### Focused transcriptomic analyses

Total RNA was extracted from mouse mouse aortas (Qiagen, RNeasy Fibrous Tissue Mini Kit) following the manufacturer’s instructions, quantified by Nanodrop, and kept frozen (-80°C) until further use. Purified RNA (250 ng/condition) was converted into cDNA by reverse transcription (RT^2^ First Strand kit, Qiagen). Atherosclerosis-focused transcriptomic analysis was then performed by quantitative PCR of the cDNA using a mouse Atherosclerosis RT^2^ Profiler PCR Array (Qiagen, Cat. # 330231, GeneGlobe ID PAMM-038Z, https://geneglobe.qiagen.com/gb/product-groups/rt2-profiler-pcr-arrays for full list and description of genes tested), following the manufacturer’s instructions. Analysis was performed automatically via the Qiagen Geneglobe analysis tool. Reference genes were selected automatically from the housekeeping gene panel and relative gene expression was calculated using the ΔΔCt method.

### Flow cytometry

Expression levels of cell surface antigens were determined by flow cytometry as previously described (Rey Nores et al., 1999). Briefly, cell suspensions (300 µl mouse peritoneal lavage or 30 µl of red-blood cell lysed mouse blood, Pharm Lyse, BD Biosciences) were prepared in sterile PBS and Fc receptors were blocked by incubation (10 min, RT) with mouse Fc block (BD Biosciences) before incubation (45 min, 4°C) with directly conjugated monoclonal antibodies (Ly6C, clone HK1.4-AF488; Ly6G, clone 1A8-AF647; CD11b, clone M1/70-PerCP Cy5.5, BioLegend). Cells were rinsed twice with PBS before immediate flow cytometry analysis on an Attune NxT or a FACS Canto II cytometer. Cellular debris and doublets were excluded based on their FSC-A/SSC-A and FSC-H/FSC-A scatter profiles, respectively. Neutrophils were defined as Ly6G^+^ and CD11b^+^ and monocytes as Ly6G^-^ and CD11b^+^. Among total monocytes, Ly6C^high^ and Ly6C ^low^ populations were determined based on their Ly6C expression. In lavage samples, peritoneal resident macrophages were defined as Ly6G- and CD11b++.

### Cell culture

Mono-Mac6 cells were cultured in RPMI 1640 medium supplemented with 10% low endotoxin fetal calf serum (FCS, HyClone; < 0.06 U/mL endotoxin), 1% insulin (Fisher Scientific), 1% Non-essential Amino Acids (Fisher Scientific) and 1% pyruvate (Fisher Scientific).

Human peripheral blood mononuclear cells (PBMC) were obtained from buffy coats (Welsh Blood Services) through Ficoll density-gradient centrifugation and cultured in RPMI 1640 medium supplemented with 10% low endotoxin FCS. Macrophages were differentiated from blood monocytes obtained by PBMC adhesion (2h, RMPI 1640 supplemented with 1% FCS). Non-adherent cells were removed, and adherent monocytes were rinsed (3 times, PBS) before culture for at least 7 days in complete medium supplemented with human M-CSF (10 ng/ml, Peprotech). The culture medium was then supplemented with fresh M-CSF every 3 days for the remainder of the culture.

### Post-infection supernatants preparation

Mono-Mac 6 cells were seeded at 800.000 cells/well in 1ml of complete medium, before stimulation or not with Heat-killed *S. epidermidis* (HK *S. epi*, 5x10^8^ cfu/ml) overnight. Bacteria-containing supernatants were then removed, cells were rinsed twice with sterile PBS and cultured for a futher 24h in 1ml complete medium before supernatant collection and filtering (0.22µm). DAMP levels in the supernatants were analysed by ELISA prior to supernatant use in functional experiments.

### Cytokine production assays

M-CSF differentiated macrophages (1.5 x 10^4^ cells/well) were cultured (18h) in the presence of the indicated concentrations of ultra-pure LPS (10 ng/ml, E. coli O111:B4 strain; Invivogen) or post-infection supernatants (50% final volume). For Calprotectin blockade, post infection supernatants or LPS were pre-incubated with Paquinimod (5µg/ml, Sigma) in culture medium (30 mins) prior to incubation with cells (18h). Cell viability was routinely assessed at the end of the experiments by Trypan Blue staining, was always > 90% and was not affected.

### Monocyte migration

Mono-Mac 6 cells (1x10^6^ cells/condition) were stimulated or not (18h) with recombinant human Calprotectin (1 µg/ml), ultra-pure LPS (10 ng/ml) or post-infection supernatants (50% final volume). For Calprotectin blockade, Calprotectin, post infection supernatants and LPS were pre-incubated with Paquinimod (5µg/ml, Sigma) in culture medium (30 mins) prior to incubation with cells (18h). Cells were then starved (1h) in serum-free medium prior to seeding (200,000 cells, in triplicates) in the top chamber of 8 µm pores transwells. The bottom compartment was filled with RPMI containing 10% FCS and recombinant human MCP-1 (CCL2, functional grade, R&D, 50ng/ml). Cell numbers were counted in the bottom compartment at the indicated time points.

### Foam cell formation

Following a starvation step (24h, no serum, RPMI 1640 supplemented with 0.2% fatty-acid free BSA), macrophages were seeded in 8-well microscopy slide (175,000 cells/well) and cultured (24h, no serum) in the presence, or not, of LDL (Invitrogen, 25 µg/ml) alone or together with ultra-pure LPS (10 ng/ml) or post-infection supernatants (50% final volume). For Calprotectin blockade, post infection supernatants and LPS were pre-incubated with Paquinimod (5µg/ml, Sigma) in RPMI without serum (30 mins) prior to incubation (24h) with macrophages in the presence of LDL. Intracellular neutral lipids were visualised by Oil Red-O staining (30 min, 37°C), as previously described (Xu et al., 2010). Slides were mounted using DAPI mounting medium (Fluoroshield, Abcam) to preserve fluorescence and visualise cell nucleus. Images (20x magnification) of 5 non-overlapping fields of view were taken for each condition on a Leica DM LA microscope. To quantify foam cell formation, 20 cells were selected from the top left corner of each image, each cell with detectable red staining was considered a foam cell, while cells without staining were considered normal cells. The process was repeated with the 5 images taken for each condition (total of 100 cells counted/condition). The percentage of foam cells per condition is shown as a measure of foam cell formation.

### Patient blood samples

Blood samples from patients on PD, before or at Day 1 of presentation with peritonitis, were collected via the Welsh Kidney Research Tissue Bank in accordance with the institutional review board of Cardiff University and the local National Health Service Research Ethics Committee (**Table 1**). Non infected baseline plasma samples were collected more than 1 week before peritonitis diagnosis. Written informed consent was obtained from all donors. Causes of CKD included focal segmental glomerulosclerosis (n=2), granulomatosis with polyangiitis, IgA nephropathy, hypertensive nephropathy, obstructive uropathy, idiopathic membranous nephropathy and congenital renal dysplasia. Samples from patients with diabetes or malignancy were excluded from the analysis. Blood was centrifuged at 1500 x g for 15 mins to obtain plasma, which was aliquoted and kept at -80°C until further use. At the time of testing, samples were thawed and kept on ice for the duration of the preparation, for analysis by ELISA according to the manufacturer’s instructions.

**Table 1:** PD patients’ characteristics at the time of non-infected plasma sample collection.

Characteristic	PD patients
Number	8
Age, yr: Median (interquartile range)	64 (33-76)
Female sex: n (%)	4 (50)
Months on PD: Median (interquartile range)	16 (1.9-25.3)
Weeks before peritonitis episode: Median (interquartile range)	11 (1-52)

### Statistical analysis

Statistical analyses were performed using GraphPad Prism (version 10) and are described in the corresponding figure legends. Normal distribution of data sets was evaluated by Shapiro Wilk test. For experiments where data from all groups was normally distributed, *p* values were calculated using a t-test (2 groups) or an ordinary one-way ANOVA followed by a Tukey test (multiple groups). In cases where data for one of more of the experimental groups being compared was not normally distributed, a Mann-Whitney U test for independent samples (2 groups) or a Kruskal-Wallis test (multiple groups) was used. For paired sample comparisons, the non-parametric Wilcoxon signed-rank test was used.

## Results

### A bacterial peritonitis episode induces vascular inflammatory and atherosclerosis-promoting responses that are long-lasting following resolution

**Figure 1:**
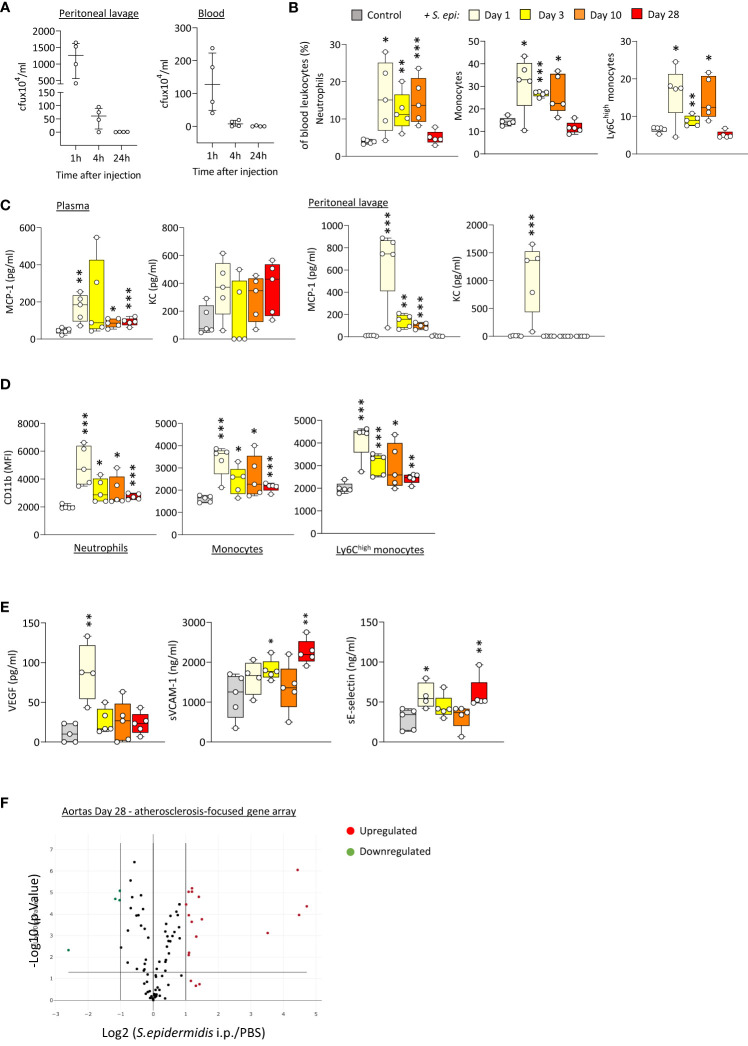
Bacterial peritonitis induces inflammatory and pro-atherosclerotic vascular responses that are long-lasting past infection clearance. C57BL/6J mice (n=5 per group) were injected intraperitoneally with live *S. epidermidis* or PBS (Control) and peritoneal lavages, blood, and aortas were obtained at the indicated time points. Bacterial numbers in blood and lavages were determined by colony counting after growth **(A)**. Innate leukocyte proportions **(B)** and CD11b expression **(D)** were determined by flow cytometry and plasma levels of cytokines **(C)** and endothelial activation markers **(E)** were quantified by ELISA. *p <0.05; **p <0.01; ***p <0.005, indicated time point vs PBS control, Mann-Whitney U test. Volcano plots **(F)** show the effect of *S. epidermidis* peritonitis on aortic atherosclerosis-associated gene expression at Day 28. Red (upregulated, fold change ≥ 2) and green (downregulated, fold change ≤ 0.5) circles represent single genes significantly affected (*p <*0.05, indicated by the horizontal line) compared to PBS control.

In order to better understand the mechanism(s) linking infection with increased long-term CV risk, peritonitis was induced in normal mice as previously described (Raby et al., 2013; Raby et al., 2017), by intraperitoneal injection of live *Staphyloccocus epidermidis* (*S. epidermidis*), a leading cause of peritonitis in PD patients (Finkelstein et al., 2002; Mujais, 2006). This model mimics the progression of a clinical bacterial peritonitis episode typically observed in PD patients. Vascular inflammatory readouts were evaluated up to 28 days following infection. While pathogen clearance from the blood and peritoneum was achieved within the first 24h post-infection (**Figure 1A**), peritonitis led to systemic and vascular inflammatory responses that were maintained for up to 28 days. Specifically, proportions of innate immune leukocytes were markedly elevated until at least Day 10, including neutrophils (CD11b^+^/Ly6G^+^), total monocytes (CD11b^+^/Ly6G^-^) and the pro-inflammatory Ly6C^high^ monocytic subset (**Figure 1B**), the latter being preferentially recruited to the atherosclerotic plaque (Tacke et al., 2007) and specifically associated with increased risk of CV events in dialysis patients (Betjes, 2013). While there was a temporary reduction in this leukocyte elevation at Day 3, probably as a result of the recruitment of these leukocyte populations to the peritoneal cavity, proportions at Day 10 had increased again, despite pathogen clearance. This was in line with the maintenance of increased plasma levels of pro-inflammatory cytokines critical to vascular inflammation and atherosclerosis progression (Boring et al., 1998; Boisvert et al., 2000; Tacke et al., 2007), notably the monocyte chemoattractant MCP-1, observed even at Day 28 (**Figure 1C**), in spite of its return to normal levels in the peritoneal cavity. A similar trend was also observed for the neutrophil chemoattractant KC in plasma (not statistically significant) and peritoneum (**Figure 1C**). In addition to their increased proportions, innate leukocytes displayed increased expression of CD11b (**Figure 1D**), an activation marker and adhesion molecule critical to leukocyte adhesion to the activated endothelium during recruitment to the inflamed intima, and these changes were maintained at Day 28. Vascular/endothelial-specific inflammatory markers indicative of endothelial cell activation (Gearing and Newman, 1993; Newman et al., 1993; Eikemo et al., 2004; Videm and Albrigtsen, 2008) were also found elevated after peritonitis (**Figure 1E**), either in short (VEGF), or longer term (soluble VCAM-1; soluble E-selectin). Together with changes in the blood, aortas from mice infected 28 days earlier displayed upregulated expression of a number of genes associated with inflammation and atherosclerosis development. Out of 84 genes tested, 15 were upregulated (fold change ≥ 2, *p* ≤ 0.05, genes indicated in red) following *S. epidermidis* administration (**Figure 1F**; **Supplementary Table 1**), including a majority of genes involved in inflammation (*Il4, Lif, Tgfb2, Tnc*), endothelial activation (*Fn1, Itga5)*, lipid metabolism and handling (*Apoa1, Apob, Hbegf*), arterial remodelling (*Ccn2, Eln*) and platelet activation (*Fga, Fgb, Serpine1, Vwf*). Of note, 4 genes were downregulated (fold change ≤ 0.5, *p* ≤ 0.05, genes indicated in green) following *S. epidermidis* peritonitis, coding notably for anti-atherosclerotic mediators, such as PPARG, a receptor whose activation leads to potent anti-atherosclerotic responses (Li and Glass, 2004; Li et al., 2004), IL-5, which slows down atherosclerosis progression (Binder et al., 2004), and ApoE, which mediates lipid clearance from plasma (Bouchareychas and Raffai, 2018).

Taken together, the *in vivo* findings demonstrated that a single peritoneal infection episode induces inflammatory systemic and vascular changes that are long-lasting, despite rapid infection clearance, and are expected to worsen atherosclerosis and CV risk.

### The long-term systemic and vascular consequences of peritonitis are maintained in chronic nephropathic mice and exacerbated in animals routinely exposed to PD solutions

PD patients display altered immune responses as a result of CKD and the PD process (Cohen et al., 2001; Anding et al., 2003; Hauser et al., 2008). Therefore, the short and long term consequences of a peritonitis episode were re-evaluated in nephropathic mice (aristolochic-acid induced nephropathy, AAN **Supplementary Figure 1A**, Lu et al., 2021; Mazzarino et al, 2023) and mice undergoing daily PD fluid (PDF) exposure following peritoneal catheter insertion (**Supplementary Figure 2A**) (Raby et al., 2018b).

**Figure 2:**
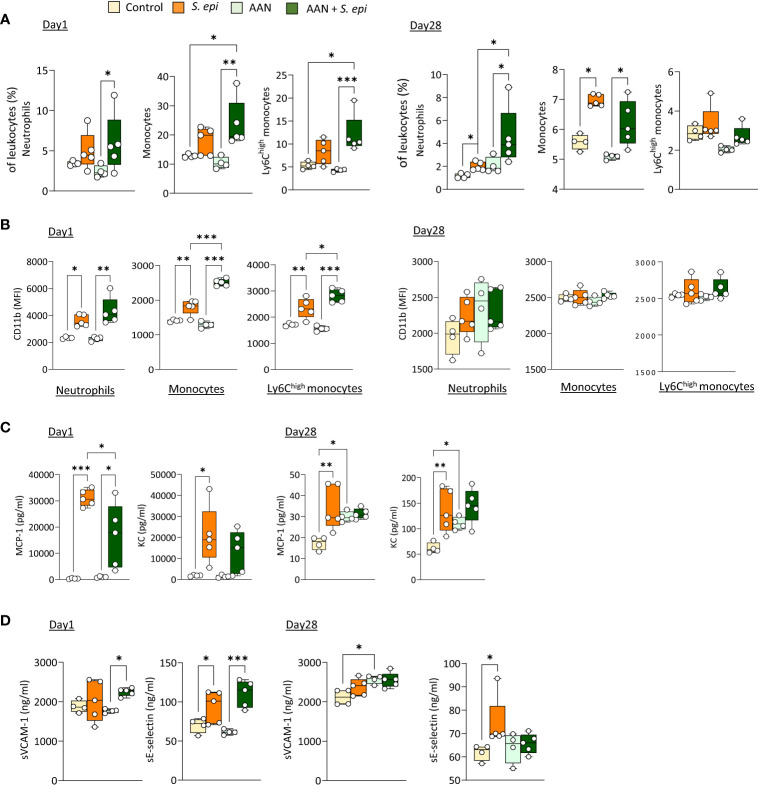
The lasting vascular responses to bacterial peritonitis are maintained in AAN mice. C57BL/6J mice (n=5/group) were i.p. injected 4 times at 3 days intervals with AA (2.5 mg/kg) or PBS. 28 days later, mice from each group were i.p. injected with *S. epidermidis* or PBS at Day 0 and culled at Day 1 or Day 28. Blood proportions of innate immune leukocytes **(A)** and adhesion molecule expression **(B)** were determined by flow cytometry and plasma levels of cytokines **(C)** and endothelial activation markers **(D)** were quantified by ELISA **(C)**. *p <*0.05; **p <0.01; ***p <0.005, ordinary one-way ANOVA (normal distribution) or Kruskal-Wallis test (non-normal distribution).

**Figure 3:**
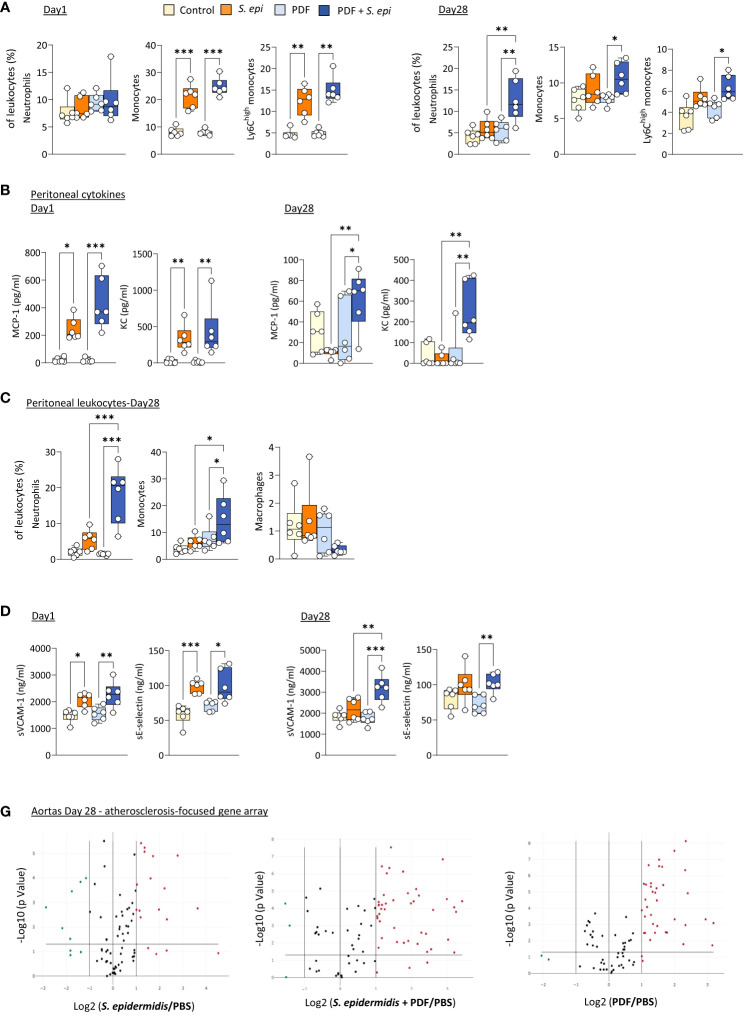
The long-term vascular responses to bacterial peritonitis are exacerbated in animals routinely exposed to PD solutions. C57BL/6 mice (n=6/group) were fitted with a peritoneal catheter, given a 7-day recovery period and instilled once daily with 2ml PBS or PDF for 14 day. Mice were then i.p. injected with *S. epidermidis* or PBS (Day 0) and culled at Day 1 or further exposed daily to PBS or PDF, prior to culling at Day 28. Peritoneal lavages, blood and aortas (Day 28 only) were collected and proportions of innate immune leukocytes in blood **(A)** and lavages **(C)** were determined by flow cytometry and plasma and peritoneal levels of cytokines **(B)** and endothelial activation markers (plasma only, **D**) were quantified by ELISA *p <0.05; **p <0.01; ***p <0.005, ordinary one-way ANOVA (normal distribution) or Kruskal-Wallis test (non-normal distribution). Volcano plots **(E)** compare the effect of *S. epidermidis*, PDF exposure + *S. epidermidis* and PDF exposure to PBS control on atherosclerosis-associated gene expression in aortas at Day 28. Red (upregulated, fold change ≥ 2) and green (downregulated, fold change ≤ 0.5) circles represent single genes significantly affected (*p <*0.05, represented by the horizontal line) compared to PBS control.

As previously described, repeated administration of AA results in nephropathy, manifested by tubular injury leading to inflammation followed by tissue remodeling and fibrosis, as well as increased creatinine plasma levels (Lu et al., 2021; Mazzarino et al, 2023 and **Supplementary Figures 1B, C**). Of note, these effects were present at the time of peritonitis and remained significant at Day 28, confirming the maintenance of kidney pathology throughout the experiment. *S. epidermidis*-infected AAN mice displayed overall innate immune leukocyte proportions similar to those observed in infected normal mice, although higher neutrophil proportions were observed at Day 28 when compared to *S. epidermidis*-infected normal mice (**Figure 2A**). Notably, CD11b was further increased in AAN infected, compared to normal infected, mice shortly after infection on total and Ly6C^high^ monocytes, when compared to *S. epidermidis*-infected normal mice (Day 1, **Figure 3B**), but this effect was lost at Day 28 (Day 28, **Figure 2B**). In contrast to the early elevation of CD11b, plasma levels of MCP-1 in AAN mice were significantly lower at Day 1 post-infection when compared to infected normal mice, and KC followed a similar trend (Day 1, **Figure 2C**). These findings are in line with the overall impaired immune cell function reported in CKD (Binder et al., 2004). While monocyte and neutrophil numbers may be higher in CKD patients, with higher levels of activation markers (e.g., CD11b), their ability to respond to pathogenic challenges, notably in terms of cytokine production, is reduced (Lim et al., 2007). Accordingly, basal levels of MCP-1 and KC were higher in AAN mice at Day 28, but the increase in response to infection was low (Day 28, **Figure 2C**). As a result, there was no difference in cytokine plasma levels between normal and AAN mice 28 days following infection. Similarly, plasma levels of solubleVCAM-1 and soluble E-selectin were not significantly different between control-infected and AAN-infected mice (**Figure 2D**), although there was an increase in sVCAM1 release in AAN mice at Day 1. Therefore, despite ongoing kidney inflammation, the inflammatory burden post-infection was not increased in AAN mice, probably in part due to a degree of immunosuppression in these animals.

Daily exposure to PDF did not affect the early (Day 1) changes in blood leukocyte proportions following peritonitis (**Figure 3A**), as leukocyte levels were similar to those observed in *S. epidermidis*-infected control mice, but it led to a long-term (Day 28) overall further increase in the proportions of all innate blood leukocytes tested, which was statistically significant for neutrophils (**Figure 3A**). Of note, CD11b expresssion levels and plasma MCP-1 and KC were unaffected by PDF exposure (**Supplementary Figures 2B, C**). However, while levels of both peritoneal MCP-1 and KC were similar in PDF and control mice shortly after infection (**Figure 3B**, Day 1), they remained elevated in PDF-exposed mice at Day 28, while they had returned to normal levels in PBS-exposed infected controls (**Figure 3B**, Day 28). Consistent with these findings, significantly higher proportions of peritoneal neutrophils and monocytes (>95% recruited monocytes were Ly6C^high^) were observed long-term (28 days) following infection, concomitant with an apparent reduction in peritoneal macrophages (**Figure 3C**). Taken together, these observations suggest that chronic PDF exposure impairs inflammation resolution following peritonitis, manifested by prolonged leukocyte recruitment to the peritoneal cavity and, consequently, to increased blood inflammatory leukocyte proportions. Interestingly, plasma sVCAM-1, but not sE-selectin, was also increased at Day 28 following peritonits in PDF-exposed, compared to control, mice. This suggests that PDF exposure also worsens long-term endothelial inflammation following peritonitis. In line with this, the combination of PDF exposure together with *S. epidermidis* peritonitis also led to an increase in the extent of inflammatory and atherosclerosis promoting genes expression in aortas at Day 28 post-infection, compared to peritonitis or PDF exposure alone (**Figure 3D**, **Supplementary Table 2**).

Thus, the lasting inflammatory vascular alterations induced by a peritonitis episode in mice were maintained during chronic nephropathy and exacerbated by chronic exposure to PDF, which can be expected to further aggravate vascular pathology following peritonitis.

### Plasma Calprotectin increases upon peritonitis in both mice and PD patients and remains elevated long after infection resolution

**Figure 4:**
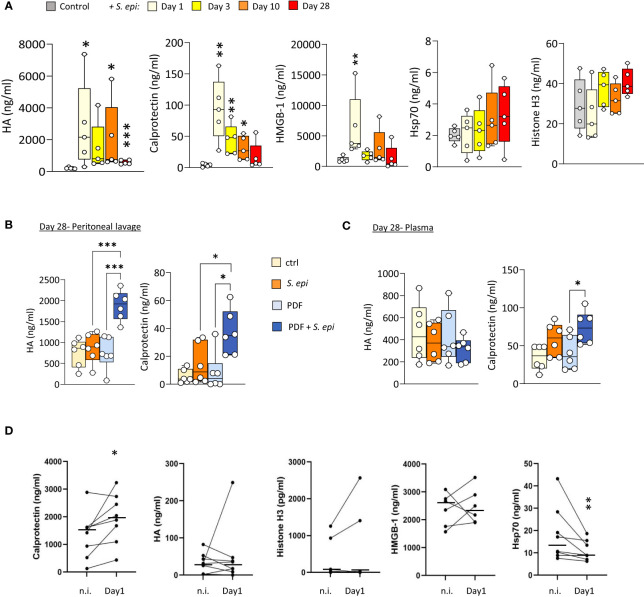
Peritonitis affects specific peripheral DAMP levels in mice and PD patients. **(A)**. C57BL/6J mice (n=5 per group) were injected intraperitoneally with live *S. epidermidis* or PBS (Control) and blood was obtained at the indicated time points. **(B, C)** C57BL/6 mice (n=6/group) were fitted with a peritoneal catheter, given a 7-day recovery period and instilled once daily with 2ml PBS or PDF for 14 days. Mice were then i.p. injected with *S. epidermidis* or PBS (Day 0) and culled at Day 1 or further exposed daily to PBS or PDF, prior to culling at Day 28. Peritoneal lavages and blood were obtained at Day 1 and Day 28. Plasma and peritoneal levels of DAMPs were determined by ELISA. *p <*0.05; **p <0.01; ***p <0.005, ordinary one-way ANOVA (normal distribution) or Kruskal-Wallis test (non-normal distribution). **(D)** Levels of the indicated DAMPs were determined by ELISA in plasma from the same patients (n=8) non-infected (n.i.) or at Day 1 of hospital admission due to peritonitis. Horizontal lines in indicate the median value for the group, symbols indicate individual data points. *p <0.05; **p <0.01 (Peritonitis vs. non-infected, n.i.), Wilcoxon signed-rank test for paired samples.

To investigate a potential role for DAMPs in inducing and maintaining peritonitis-induced vascular inflammatory responses in the long term, plasma levels of DAMPs were investigated first in mice before, during, and after *S. epidermidis*-induced peritonitis. Five DAMPs known to be ligands of Toll-like receptors (TLR) were selected to encompass well-described DAMP-families involved in inflammatory pathologies (Roh and Sohn, 2018). Plasma Calprotectin and Hyaluronic acid (HA) levels were significantly increased at Day 1 following infection and decreased over time but remained mostly over the normal background levels at Day 28 (**Figure 4A**). By contrast, levels of HMGB-1 were elevated at Day 1 but returned to normal by Day 3, while Hsp70 and Histone H3 were mainly unaffected (**Figure 4A**).

Of particular relevance to peritonitis in PD, routine exposure to PDF led to the maintenance of higher peritoneal levels of Calprotectin and HA at Day 28 post-infection (**Figure 4B**), compared to control mice, and a slight increase of Calprotectin, but not HA, in plasma (**Figure 4C**). This is in line with the overall increase in blood and peritoneal leukocyte proportions seen at Day 28 in PDF infected mice, and suggests that increased DAMP release may be involved in the worsening effect of PDF exposure on long-term vascular inflammation post-peritonitis described above.

Blood transcriptomic analysis of *S. epidermidis*-infected mice revealed that expression of the two genes coding for Calprotectin subunits, *S100a8* and *S100a9*, was strongly upregulated shortly after peritonitis and remained so, although to a lesser extent, at Day28 (**Table 2**). This suggests that the observed increase in plasma Calprotectin following peritonitis is driven by a lasting increase in the expression of its genes by blood leukocytes. Expression of the genes coding for HA synthases (*Has1-3*) and hyaluronidases (*Hyal1, 3*) was not significantly affected by peritonitis. There was some increase in hyaluronidase 1 expression at Day 28 (**Table 2**), which may suggest increased breakdown of large HA into the smaller pro-inflammatory DAMP forms.

**Table 2:** Effect of *S. epidermidis* peritonitis in mice on blood gene expression of Calprotectin, Hsp70 and HA synthases and hyaluronases*.

	Day 3		Day 28	
Gene Symbol	Fold Change	p	Fold Change	p
*Has1*	0.9	*0.89*	0.6	*0.32*
*Has2*	2.0	*0.64*	0.2	*0.39*
*Has3*	0.9	*0.92*	4.9	*0.15*
*Hyal1*	1.0	*0.96*	1.8	*0.10*
*Hyal3*	1.7	*0.13*	1.6	*0.19*
*S100a8*	44.3	*<0.005*	6.3	*<0.005*
*S100a9*	32.4	*<0.005*	4.0	*<0.005*

*In comparison to PBS control group.

To verify the clinical relevance of the findings made in mice, DAMP levels were determined in PD patients’ plasma before and during a peritoneal infection. Despite significant patient heterogeneity in DAMP levels, Calprotectin was found significantly elevated during peritonitis, (**Figure 4D**). Plasma levels of most other DAMPs tested were not consistently affected by peritonitis, while Hsp70 showed a decrease (**Figure 4D**).

Therefore, the relevance of Calprotectin to the maintenance of systemic and vascular inflammation following infection was investigated further *in vitro* and *in vivo*.

### Calprotectin is an important contributor to increased monocyte chemotaxis and foam cell formation upon-infection

Our recent findings demonstrated the ability of purified Calprotectin to promote *in vitro* a number of key cellular functions critical to vascular inflammation, endothelial dysfunction and atherosclerosis development including decreased trans-endothelial resistance, increased monocyte chemotaxis and increased foam cell formation (Mazzarino M et al., 2023).

**Figure 5:**
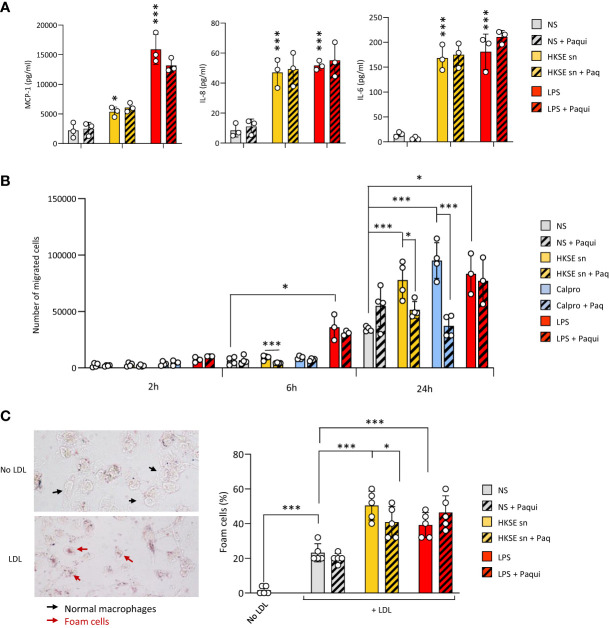
Culture supernatants from infected cells promote monocyte chemotaxis and macrophage foam cell formation in a Calprotectin-dependent manner. **(A, B)** Triplicate cultures of monocyte-derived macrophages **(A)** or Mono-Mac 6 monocytes **(B)** were stimulated (18h, 37°C), or not (NS), with LPS (10 ng/ml), Heat-killed *S. epidermis* post-infection supernatants (50% of final volume) or purified Calprotectin (1µg/ml), in the presence or absence of Paquinimod (5 µg/ml). Cytokine levels were determined by ELISA in macrophage culture supernatants **(A)** and Mono-Mac 6 migration towards MCP-1 was evaluated following starving in serum-free medium (1h) prior to seeding (200,000 cells, in triplicates) in the top chamber of 8 µm pores trans-wells **(B)**. **(C)** Monocyte-derived macrophages were exposed (24h) to low-density lipoprotein (LDL) (25 µg/ml), alone or together with LPS (200 ng/ml), HKSE post-infection supernatants (50% of final volume) or purified Calprotectin (1µg/ml), in the presence or absence of Paquinimod (5 µg/ml) prior to staining with Oil Red-O for lipid visualisation by light microscopy (representative images shown). Plots show the percentage of foam cells in each condition. Results are shown as mean (+/- SD) of 3 experiments. *p <0.05; ***p <0.005 (A, vs NS control, B-C, as indicated), ordinary one-way ANOVA.

To verify the contribution of Calprotectin to these key processes when it is part of a mix of mediators released by cells following pathogen encounter, DAMP-containing post-infection supernatants were generated by monocyte exposure to Heat-killed *S. epidermidis* (**Supplementary Figure 3A**). These supernatants contained Calprotectin as well as other DAMPs released following bacterial encounter, notably Hsp70 but not HA (**Supplementary Figure 3B**). The contribution of Calprotectin to the supernatant-induced responses was assessed by testing the effect of the pharmacological inhibitor of Calprotectin, Paquinimod, which interacts specifically with the S100A9 subunit and blocks its recognition by TLR4 (Bjork et al., 2009; Bengtsson et al., 2012). Cytokine release by macrophages stimulated with post-infection supernatants was not inhibited by addition of Paquinimod (**Figure 5A**), suggesting that other factors (other DAMPs, remaining bacterial components, inflammatory cytokines) in the post-infection supernatants are bigger contributors to the release of these pro-inflammatory cytokines. Interestingly, the supernatants’ ability to promote monocyte migration towards MCP-1 (**Figure 5B**) as well as macrophage-to-foam cell transition (**Figure 5C**), two critical mechanisms driving the atherosclerosis process (Ilhan and Kalkanli, 2015; Boring et al., 1998; Tacke et al., 2007), were significantly inhibited by Calprotectin blockade.

Thus, blockade of infection-induced Calprotectin indicated that it differentially promotes cellular responses that are critical to initiating and maintaining vascular inflammation and atherosclerosis, and pointed at this DAMP as an important CV pathology-inducing factor released by cells following pathogen encounter.

### Calprotectin blockade inhibits the short and long-term systemic and vascular consequences of bacterial-induced peritoneal inflammation in mice

**Figure 6:**
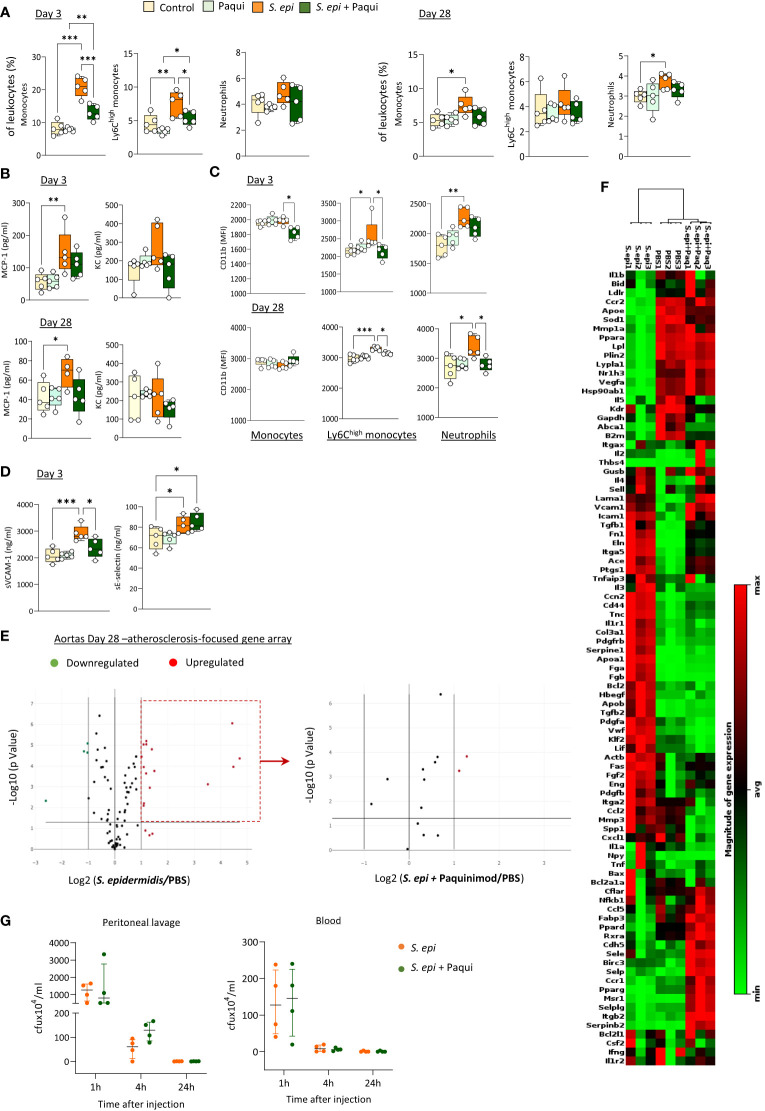
Calprotectin blocking inhibits peritonitis-induced systemic inflammatory and pro-atherosclerotic responses *in vivo.* C57BL/6J mice (n=5 per group) were injected intraperitoneally with live *S. epidermidis* or PBS (Control), in the presence or absence of Paquinimod (1mg/kg), and Paquinimod administration was repeated weekly thereafter. Peritoneal lavages, blood, and aortas were obtained at the indicated time points. Blood innate leukocyte proportions **(A)** and CD11b expression levels **(B)** were determined by flow cytometry at Day 3 and Day 28 and plasma levels of cytokines **(C)** and endothelial activation markers **(D)** were quantified by ELISA. Bacterial counts in plasma and lavages were determined by growth on agar plates 1h, 4h and 24h post-infection **(G)**. *p <0.05; **p <0.01; ***p <0.005, ordinary one-way ANOVA (normal distribution) or Kruskal-Wallis test (non-normal distribution). Volcano plots **(E)** compare the effect of *S. epidermidis* and *S. epidermidis* + Paquinimod on atherosclerosis-associated gene expression in aortas at Day 21. Focused volcano plot (right) shows the effect of Paquinimod on the *S. epidermidis* upregulated genes. Red (upregulated, fold change ≥ 2) and green (downregulated, fold change ≤ 0.5) circles represent single genes significantly affected (*p <*0.05, represented by the horizontal line) compared to PBS control. Heatmap in **(F)** displays experimental group hierarchical clustering, as determined according to the aortic expression levels of the 84 genes tested. Each column represents a sample; each row represents a gene; the relative gene expression scale is depicted on the right.

To determine the extent of Calprotectin’s contribution *in vivo* to the initiation and maintenance of peripheral and vascular inflammation following peritonitis, the pharmacological inhibitor of Calprotectin, Paquinimod, which interacts specifically with the S100A9 subunit and blocks its recognition by PRR (Bengtsson et al., 2012), was administered to mice at the time of peritonitis induction and repeated weekly thereafter. *S. epidermidis-*peritonitis resulted in a significant rise in the proportion of total blood monocytes at both Day 3 and Day 28, and Ly6C^high^ monocytes at Day 3, whereas neutrophils were increased at Day 28. Calprotectin blockade inhibited these effects (**Figure 6A**). Similarly, Paquinimod also prevented the increase in plasma MCP-1 (**Figure 6B**) and reversed the peritonitis-induced increase in CD11b expression (**Figure 6C**) and sVCAM-1 plasma levels, but not that of sE-selectin (**Figure 6D**).

In addition to its systemic anti-inflammatory effect, Calprotectin blockade had a profound protective effect against peritonitis-induced aortic overexpression of pro-atherosclerotic genes. Paquinimod downregulated the aortic expression of all pro-inflammatory and pro-atherosclerotic genes overexpressed at Day 28 following peritonitis (**Figure 6D**; **Supplementary Table 3**) Specifically, the expression of 13 out of the 15 genes found up-regulated at this time point was reduced back to normal levels by Calprotectin blockade, while 2 (*Apo1a, Fga*) were reduced but remained elevated compared to control mice. Blocking of Calprotectin also reversed changes in expression of 4 downregulated genes, bringing 3 back to normal levels and halving the peritonitis-induced inhibition of *Il5* expression. Clustering analysis (**Figure 6E**) revealed that the aortic inflammatory and atherosclerosis-associated gene expression profile of Day 28 peritonitis mice administered with Paquinimod was significantly closer to that of control than to peritonitis animals.

Thus, Calprotectin blockade effectively reduced peritonitis-induced key mediators of chronic vascular inflammation and pathology in the short and long-term following infection, demonstrating the substantial contribution of this DAMP to these processes. Critically, Paquinimod’s anti-inflammatory effect did not compromise the ability of mice to clear the peritonitis infection, both from the infection site (peritoneal lavage) and the blood (**Figure 6F**).

## Discussion

Bacterial infections have been associated with increased long term CV risk (Chen et al., 2012; Bergh et al., 2017) and both serious and less severe local infections were also found to increase this risk (Jafarzadeh et al., 2016; Sanz et al., 2020; Zardawi et al., 2020), notably via the promotion of atherosclerosis (Beukers et al., 2017). In CKD patients on PD, the risk of CV death is up to 10 times higher than in the general population and further increases following each episode of infectious peritonitis (Ye et al., 2017; Pecoits-Filho et al., 2018).

Increasing evidence suggests that infection-induced inflammation, rather than the pathogen itself, promotes CV pathologies (Bergh et al., 2017; Pecoits-Filho et al., 2018). However, the mechanism(s) by which infection-induced inflammation is maintained systemically and in the vessels in the long-term after infection resolution are not well understood, therefore effective and safe anti-inflammatory strategies to reduce it remain to be developed.

This study revealed that a single peritoneal infection episode, cleared within 24h, led to systemic and vascular inflammatory responses that are expected to worsen CV risk and were maintained for at least 28 days in mice. These included increased blood proportions of total and Ly6C^high^ inflammatory monocytes and neutrophils, which displayed higher levels of the adhesion molecule CD11b, higher plasma levels of cytokines as wells of markers indicative of endothelial activation, together with increased pro-inflammatory and atherosclerotic gene expression in aortas. Of particular relevance to PD patients, these effects were also observed in nephropathic mice and were further increased in mice exposed to PD solutions daily. Critically, the DAMP Calprotectin was found substantially involved in these processes.

The therapeutic potential of targeting DAMPs to reduce chronic vascular inflammation following an infection was first highlighted by the increased plasma levels of Calprotectin and HA, maintained over 28 days, after a peritonitis episode in mice. Plasma levels of Calprotectin, but not HA or the other DAMPs tested, were also found elevated in PD patients with ongoing peritonitis, in line with findings in other non-PD associated bacterial infection scenarios (Havelka et al., 2020; Lamot et al., 2022). In steady state, Calprotectin is an intracellular Ca^2+^-binding protein, but is released in large amounts upon cell activation, notably by phagocytes (Hessian et al., 1993). This study has not investigated the cellular source(s) of Calprotectin, but it can be speculated that elevated Calprotectin plasma levels come from neutrophils and monocytes to a large extent, as they are the main source in blood (Hessian et al., 1993). Extracellular Calprotectin acts as a DAMP by engaging both TLR4 and RAGE (Schiopu and Cotoi, 2013) and also contribute to the response to infection via direct anti-microbial properties, notably, but not exclusively, due to its ability to withhold transition metals from pathogens (Sohnle et al., 2000; Besold et al., 2018).

Increased Calprotectin levels in the blood and at the atherosclerotic plaque site have been shown to correlate with increased risk of CV events, in the normal population (Kunutsor et al., 2018; Roh and Sohn, 2018) and in CKD and PD patients (Amaya-Garrido et al., 2023; Mazzarino M et al., 2023). While Calprotectin was recently shown to promote vascular inflammation and calcification in animals with CKD, the extent of its contribution to promoting vascular disease following an infection has remained untested. The experiments using supernatants from infected monocytes combined with the Calprotectin inhibitor Paquinimod presented here demonstrated that, among the mix of mediators released by the cells following pathogen encounter, including bacterial components that may remain after infection clearance, Calprotectin is a major contributor to the observed increase in monocyte chemotaxis and foam cell formation, two key processes in vascular inflammation and atherosclerosis progression. Of note the post-infection culture supernatants were prepared from monocytes and not a mix of leukocyte populations, however, this model provides the proof of concept of the importance of post-infection Calprotectin release, as these cells are average producers of Calprotectin, releasing less than neutrophils but more than lymphocytes (Hessian et al., 1993).

The potential of Calprotectin as a therapeutic target to reduce CV risk following an infection was further demonstrated by the ability of Paquinimod to prevent the lasting dysregulated systemic and vascular inflammatory responses and pro-atherosclerotic gene expression that followed peritonitis in mice. The effect of Calprotectin blockade was robust and reduced both early and late systemic and vascular responses. Although Paquinimod treatment was not carried out in PDF-exposed mice, it is reasonable to expect that this strategy will also be efficient in this condition, as the increased long-term vascular responses to peritonitis in PDF-exposed mice were associated with further increased peritoneal and plasma Calprotectin levels, suggesting that it remains a valid therapeutic target in this model. A different optimal dose of Paquinimod may be necessary in this case to achieve similar levels of inhibition.

Mouse models of hyperlipidemia-induced full-blown atherosclerosis (e.g., ApoE^-/-^ or LDLR^-/-^ mice on a high-fat diet) were not used here, since, as opposed to the general population, atherosclerosis is often found without hyperlipidemia in late CKD patients (Massy and de Zeeuw, 2013; Ridker et al., 2017), in line with the lack of statin protection in this population. Thus, while peritonitis induced a range of long-term inflammatory and atherosclerosis-promoting responses in blood and aortas, full-scale atherosclerosis was not evaluated. However, it is reasonable, to expect that plaque burden would be worsened by maintained increased vascular inflammation and arterial atherosclerosis-associated gene expression following an infection, and that it would be ameliorated following inhibition of these responses by Paquinimod.

Because TLR involvement in CVD progression, in particular atherosclerosis, is well established (Ionita et al., 2010; Roshan et al., 2016; Bezhaeva et al., 2022), we have limited our analysis to DAMPs that are described as agonists of at least one TLR, e.g., Calprotectin, a TLR4 ligand. Direct Calprotectin inhibition was however selected over a TLR4 blocking strategy, as diminished TLR4 activity has long been associated with impaired bacterial and viral infection clearance (O’Brien et al., 1980; Woods et al., 1988; Fukusaki et al., 2007; Khanolkar et al., 2009; Mansur et al., 2014). By contrast, the administration of Paquinimod during and after *S. epidermidis* peritonitis was demonstrated here to not compromise bacterial clearance, in line with observations made in other bacterial and fungal infections models (Masouris et al., 2017; Shankar et al., 2021) This is particularly relevant when considering the long-term management of an ongoing infection, and in PD patients, who are known to be susceptible to recurrent infections. In addition, it is possible that Calprotectin’s effect is in part mediated by its other PRR, RAGE (Bjork et al., 2009; Yan et al., 2013), pointing at direct Calprotectin targeting as a potentially better strategy than PRR blockade.

In addition to CVD, bacterial infections also have a long term worsening effect on a number of other inflammatory pathologies such as Alzheimer’s disease (Seaks and Wilcock, 2020) and rheumatoid arthritis (Li et al., 2013). While the role of DAMPs in general, and Calprotectin in particular, in mediating these effects has not been elucidated, the findings presented here suggest that reducing long-term peripheral inflammation following an infection by targeting Calprotectin may also be a promising approach to reduce the worsening effects of bacterial infections on these pathologies.

## Data availability statement

The datasets presented in this study can be found in online repositories. The names of the repository/repositories and accession number(s) can be found here: NCBI GEO, accession numbers: GSE246100, GSE246101 and GSE246102.

## Ethics statement

The studies involving humans were approved by Wales Kidney Research Tissue Bank. The studies were conducted in accordance with the local legislation and institutional requirements. The participants provided their written informed consent to participate in this study. The animal study was approved by Animal Welfare Ethical Review Body (AWERB) of Cardiff University. The study was conducted in accordance with the local legislation and institutional requirements.

## Author contributions

EC: Conceptualization, Data curation, Formal analysis, Investigation, Visualization, Writing – review & editing. MM: Formal analysis, Investigation, Writing – review & editing. GG-M: Supervision, Writing – review & editing, Investigation, Methodology. VK: Investigation, Writing – review & editing. SM: Supervision, Writing – review & editing. DF: Funding acquisition, Supervision, Writing – review & editing. ML-C: Methodology, Supervision, Writing – review & editing, Funding acquisition. MOL: Supervision, Writing – review & editing, Conceptualization, Validation, Writing – original draft, Formal analysis. A-CR:Investigation, Writing – review & editing, Conceptualization, Data curation, Formal analysis, Funding acquisition, Methodology, Supervision, Validation, Visualization, Writing – original draft.

## References

[B1] Amaya-Garrido A. Brunet M. Buffin-Meyer B. Piedrafita A. Grzesiak L. Agbegbo E.. (2023). Calprotectin is a contributor to and potential therapeutic target for vascular calcification in chronic kidney disease. Sci. Trans. Med. 15, eabn5939. doi: 10.1126/scitranslmed.abn5939 37672568

[B2] Anding K. Gross P. Rost J. M. Allgaier D. Jacobs E. (2003). The influence of uraemia and haemodialysis on neutrophil phagocytosis and antimicrobial killing. Nephrol. Dialysis Transplant. 18, 2067–2073. doi: 10.1093/ndt/gfg330 13679482

[B3] Bengtsson A. A. Sturfelt G. Lood C. Ronnblom L. van Vollenhoven R. F. Axelsson B.. (2012). Pharmacokinetics, tolerability, and preliminary efficacy of paquinimod (ABR-215757), a new quinoline-3-carboxamide derivative: studies in lupus-prone mice and a multicenter, randomized, double-blind, placebo-controlled, repeat-dose, dose-ranging study in patients with systemic lupus erythematosus. Arthritis Rheum 64, 1579–1588. doi: 10.1002/art.33493 22131101

[B4] Bergh C. Fall K. Udumyan R. Sjöqvist H. Fröbert O. Montgomery S. (2017). Severe infections and subsequent delayed cardiovascular disease. Eur. J. Prev. Cardiol. 24, 1958–1966. doi: 10.1177/2047487317724009 28764553

[B5] Besold A. N. Culbertson E. M. Nam L. Hobbs R. P. Boyko A. Maxwell C. N.. (2018). Antimicrobial action of calprotectin that does not involve metal withholding. Metallomics 10, 1728–1742. doi: 10.1039/c8mt00133b 30206620 PMC6417507

[B6] Betjes M. G. (2013). Immune cell dysfunction and inflammation in end-stage renal disease. Nat. Rev. Nephrol. 9, 255–265. doi: 10.1038/nrneph.2013.44 23507826

[B7] Beukers N. G. van der Heijden G. J. van Wijk A. J. Loos B. G. (2017). Periodontitis is an independent risk indicator for atherosclerotic cardiovascular diseases among 60 174 participants in a large dental school in the Netherlands. J. Epidemiol. Community Health 71, 37–42. doi: 10.1136/jech-2015-206745 27502782 PMC5256268

[B8] Bezhaeva T. Karper J. Quax P. H. A. de Vries M. R. (2022). The intriguing role of TLR accessory molecules in cardiovascular health and disease. Front. Cardiovasc. Med. 9. doi: 10.3389/fcvm.2022.820962 PMC8884272 35237675

[B9] Binder C. J. Hartvigsen K. Chang M. K. Miller M. Broide D. Palinski W.. (2004). IL-5 links adaptive and natural immunity specific for epitopes of oxidized LDL and protects from atherosclerosis. J. Clin. Invest. 114, 427–437. doi: 10.1172/JCI20479 15286809 PMC484976

[B10] Bjork P. Bjork A. Vogl T. Stenstrom M. Liberg D. Olsson A.. (2009). Identification of human S100A9 as a novel target for treatment of autoimmune disease via binding to quinoline-3-carboxamides. PloS Biol. 7, e97. doi: 10.1371/journal.pbio.1000097 19402754 PMC2671563

[B11] Boisvert W. A. Curtiss L. K. Terkeltaub R. A. (2000). Interleukin-8 and its receptor CXCR2 in atherosclerosis. Immunol. Res. 21, 129–137. doi: 10.1385/ir:21:2-3:129 10852110

[B12] Boring L. Gosling J. Cleary M. Charo I. F. (1998). Decreased lesion formation in CCR2-/- mice reveals a role for chemokines in the initiation of atherosclerosis. Nature 394, 894–897. doi: 10.1038/29788 9732872

[B13] Bouchareychas L. Raffai R. L. (2018). Apolipoprotein E and atherosclerosis: from lipoprotein metabolism to microRNA control of inflammation. J. Cardiovasc. Dev. Dis. 5, 30. doi: 10.3390/jcdd5020030 29789495 PMC6023389

[B14] Cheikh Hassan H. I. Murali K. Lonergan M. Boudville N. Johnson D. Borlace M.. (2022). Association of peritonitis with cardiovascular mortality over time in the peritoneal dialysis population: an Australia and New Zealand dialysis and transplant registry study. Kidney Int. Rep. 7, 2388–2396. doi: 10.1016/j.ekir.2022.08.008 36531876 PMC9751840

[B15] Chen L. F. Chen H. P. Huang Y. S. Huang K. Y. Chou P. Lee C. C. (2012). Pneumococcal pneumonia and the risk of stroke: a population-based follow-up study. PloS One 7, e51452. doi: 10.1371/journal.pone.0051452 23251538 PMC3520842

[B16] Clayton T. C. Thompson M. Meade T. W. (2007). Recent respiratory infection and risk of cardiovascular disease: case-control study through a general practice database. Eur. Heart J. 29, 96–103. doi: 10.1093/eurheartj/ehm516 18063596

[B17] Cohen G. Rudnicki M. Hörl W. H. (2001). Uremic toxins modulate the spontaneous apoptotic cell death and essential functions of neutrophils. Kidney Int. 59, S48–S52. doi: 10.1046/j.1523-1755.2001.59780048.x 11168982

[B18] Dalager-Pedersen M. Sogaard M. Schonheyder H. C. Nielsen H. Thomsen R. W. (2014). Risk for myocardial infarction and stroke after community-acquired bacteremia: a 20-year population-based cohort study. Circulation 129, 1387–1396. doi: 10.1161/CIRCULATIONAHA.113.006699 24523433

[B19] Ding Z. Du F. Averitt V. R. Jakobsson G. Ronnow C. F. Rahman M.. (2021). Targeting S100A9 reduces neutrophil recruitment, inflammation and lung damage in abdominal sepsis. Int. J. Mol. Sci. 22, 12923. doi: 10.3390/ijms222312923 34884728 PMC8658007

[B20] Eikemo H. Sellevold O. F. Videm V. (2004). Markers for endothelial activation during open heart surgery. Ann. Thorac. Surg. 77, 214–219. doi: 10.1016/s0003-4975(03)01060-9 14726064

[B21] Feldman N. Rotter-Maskowitz A. Okun E. (2015). DAMPs as mediators of sterile inflammation in aging-related pathologies. Ageing Res. Rev. 24, 29–39. doi: 10.1016/j.arr.2015.01.003 25641058

[B22] Finkelstein E. S. Jekel J. Troidle L. Gorban-Brennan N. Finkelstein F. O. Bia F. J. (2002). Patterns of infection in patients maintained on long-term peritoneal dialysis therapy with multiple episodes of peritonitis. Am. J. Kidney Dis. 39 1278–1286. doi: 10.1053/ajkd.2002.33403 12046042

[B23] Fukusaki T. Ohara N. Hara Y. Yoshimura A. Yoshiura K. (2007). Evidence for association between a Toll-like receptor 4 gene polymorphism and moderate/severe periodontitis in the Japanese population. J. Periodontal Res. 42, 541–545. doi: 10.1111/j.1600-0765.2007.00979.x 17956467

[B24] Gansevoort R. T. Correa-Rotter R. Hemmelgarn B. R. Jafar T. H. Heerspink H. J. Mann J. F.. (2013). Chronic kidney disease and cardiovascular risk: epidemiology, mechanisms, and prevention. Lancet 382, 339–352. doi: 10.1016/S0140-6736(13)60595-4 23727170

[B25] Gearing A. J. Newman W. (1993). Circulating adhesion molecules in disease. Immunol. Today 14, 506–512. doi: 10.1016/0167-5699(93)90267-O 7506035

[B26] Gonzalez-Mateo G. T. Loureiro J. Jimenez-Hefferman J. A. Bajo M. A. Selgas R. Lopez-Cabrera M.. (2009). Chronic exposure of mouse peritoneum to peritoneal dialysis fluid: structural and functional alterations of the peritoneal membrane. Perit Dial Int. 29, 227–230. doi: 10.1177/089686080902900218 19293361

[B27] Hauser A. B. Stinghen A. E. M. Kato S. Bucharles S. Aita C. Yuzawa Y.. (2008). Characteris tics and causes of immune dysfunction related to uremia and dialysis. Peritoneal Dialysis Int. 28, 183–187. doi: 10.1177/089686080802803s34 18552253

[B28] Havelka A. Sejersen K. Venge P. Pauksens K. Larsson A. (2020). Calprotectin, a new biomarker for diagnosis of acute respiratory infections. Sci. Rep. 10, 4208. doi: 10.1038/s41598-020-61094-z 32144345 PMC7060262

[B29] Hessian P. A. Edgeworth J. Hogg N. (1993). MRP-8 and MRP-14, two abundant Ca(2+)-binding proteins of neutrophils and monocytes. J. Leukoc. Biol. 53, 197–204. doi: 10.1002/jlb.53.2.197 8445331

[B30] Ilhan F. Kalkanli S. T. (2015). Atherosclerosis and the role of immune cells. World J. Clin. cases 3, 345–352. doi: 10.12998/wjcc.v3.i4.345 25879006 PMC4391004

[B31] Ionita M. G. Arslan F. de Kleijn D. P. Pasterkamp G. (2010). Endogenous inflammatory molecules engage Toll-like receptors in cardiovascular disease. J. innate Immun. 2, 307–315. doi: 10.1159/000314270 20431283

[B32] Jafarzadeh S. R. Thomas B. S. Warren D. K. Gill J. Fraser V. J. (2016). Longitudinal study of the effects of bacteremia and sepsis on 5-year risk of cardiovascular events. Clin. Infect. Dis. 63, 495–500. doi: 10.1093/cid/ciw320 27193746 PMC4967600

[B33] Khanolkar A. Hartwig S. M. Haag B. A. Meyerholz D. K. Harty J. T. Varga S. M. (2009). Toll-like receptor 4 deficiency increases disease and mortality after mouse hepatitis virus type 1 infection of susceptible C3H mice. J. Virol. 83, 8946–8956. doi: 10.1128/JVI.01857-08 19553337 PMC2738158

[B34] Kunutsor S. K. Flores-Guerrero J. L. Kieneker L. M. Nilsen T. Hidden C. Sundrehagen E.. (2018). Plasma calprotectin and risk of cardiovascular disease: Findings from the PREVEND prospective cohort study. Atherosclerosis 275, 205–213. doi: 10.1016/j.atherosclerosis.2018.06.817 29957458

[B35] Lamot M. Miler M. Nikolac Gabaj N. Lamot L. Milosevic M. Harjacek M.. (2022). Serum calprotectin is a valid biomarker in distinction of bacterial urinary tract infection from viral respiratory illness in children under 3 years of age. Front. Pediatr. 10. doi: 10.3389/fped.2022.768260 PMC8964143 35359908

[B36] Li A. C. Binder C. J. Gutierrez A. Brown K. K. Plotkin C. R. Pattison J. W.. (2004). Differential inhibition of macrophage foam-cell formation and atherosclerosis in mice by PPARalpha, beta/delta, and gamma. J. Clin. Invest. 114, 1564–1576. doi: 10.1172/JCI18730 15578089 PMC529277

[B37] Li A. C. Glass C. K. (2004). PPAR- and LXR-dependent pathways controlling lipid metabolism and the development of atherosclerosis. J. Lipid Res. 45, 2161–2173. doi: 10.1194/jlr.R400010-JLR200 15489539

[B38] Li S. Yu Y. Yue Y. Zhang Z. Su K. (2013). Microbial infection and rheumatoid arthritis. J. Clin. Cell Immunol. 4, 174. doi: 10.4172/2155-9899.1000174 25133066 PMC4131749

[B39] Lim W. H. Kireta S. Leedham E. Russ G. R. Coates P. T. (2007). Uremia impairs monocyte and monocyte-derived dendritic cell function in hemodialysis patients. Kidney Int. 72, 1138–1148. doi: 10.1038/sj.ki.5002425 17728708

[B40] Liu M. Li X. C. Lu L. Cao Y. Sun R. R. Chen S.. (2014). Cardiovascular disease and its relationship with chronic kidney disease. Eur. Rev. Med. Pharmacol. Sci. 18, 2918–2926. 25339487

[B41] Lu Y. A. Liao C. T. Raybould R. Talabani B. Grigorieva I. Szomolay B.. (2021). Single-nucleus RNA sequencing identifies new classes of proximal tubular epithelial cells in kidney fibrosis. J. Am. Soc. Nephrol. 32, 2501–2516. doi: 10.1681/ASN.2020081143 34155061 PMC8722798

[B42] Mansur A. von Gruben L. Popov A. F. Steinau M. Bergmann I. Ross D.. (2014). The regulatory toll-like receptor 4 genetic polymorphism rs11536889 is associated with renal, coagulation and hepatic organ failure in sepsis patients. J. Transl. Med. 12, 177. doi: 10.1186/1479-5876-12-177 24950711 PMC4085654

[B43] Masouris I. Klein M. Dyckhoff S. Angele B. Pfister H. W. Koedel U. (2017). Inhibition of DAMP signaling as an effective adjunctive treatment strategy in pneumococcal meningitis. J. Neuroinflamm. 14, 214. doi: 10.1186/s12974-017-0989-0 PMC5669003 29096648

[B44] Massy Z. A. de Zeeuw D. (2013). LDL cholesterol in CKD–to treat or not to treat? Kidney Int. 84, 451–456. doi: 10.1038/ki.2013.181 23698234

[B45] Mazzarino M C. E. Bartosova M. Marinovic I I. N. Hughes T. R. Schmitt CP R. D. Labeta M. O.. (2023). Therapeutic targeting of chronic kidney disease-associated DAMPs differentially contributing to vascular pathology. Front. Immunol. 14. doi: 10.3389/fimmu.2023.1240679 PMC10577224 37849759

[B46] Mujais S. (2006). Microbiology and outcomes of peritonitis in North America. Kidney Int. Suppl. 55–62. doi: 10.1038/sj.ki.5001916 17080112

[B47] Newman W. Beall L. D. Carson C. W. Hunder G. G. Graben N. Randhawa Z. I.. (1993). Soluble E-selectin is found in supernatants of activated endothelial cells and is elevated in the serum of patients with septic shock. J. Immunol. 150, 644–654. doi: 10.4049/jimmunol.150.2.644 7678280

[B48] O’Brien A. D. Rosenstreich D. L. Scher I. Campbell G. H. MacDermott R. P. Formal S. B. (1980). Genetic control of susceptibility to Salmonella typhimurium in mice: role of the LPS gene. J. Immunol. 124, 20–24. doi: 10.4049/jimmunol.124.1.20 6985638

[B49] Parke A. Unge C. Yu D. Sunden-Cullberg J. Stralin K. (2023). Plasma calprotectin as an indicator of need of transfer to intensive care in patients with suspected sepsis at the emergency department. BMC Emerg. Med. 23, 16. doi: 10.1186/s12873-023-00785-y 36774492 PMC9922172

[B50] Pecoits-Filho R. Yabumoto F. M. Campos L. G. Moraes T. P. Figueiredo A. E. Olandoski M.. (2018). Peritonitis as a risk factor for long-term cardiovascular mortality in peritoneal dialysis patients: The case of a friendly fire? Nephrol. (Carlton) 23, 253–258. doi: 10.1111/nep.12986 28010053

[B51] Raby A. C. Colmont C. S. Kift-Morgan A. Kohl J. Eberl M. Fraser D.. (2017). Toll-like receptors 2 and 4 are potential therapeutic targets in peritoneal dialysis-associated fibrosis. J. Am. Soc. Nephrol. 28, 461–478. doi: 10.1681/ASN.2015080923 27432741 PMC5280005

[B52] Raby A.-C. González-Mateo G. T. Williams A. Topley N. Fraser D. López-Cabrera M.. (2018a). Targeting Toll-like receptors with soluble Toll-like receptor 2 prevents peritoneal dialysis solution–induced fibrosis. Kidney Int. 94, 346–362. doi: 10.1016/j.kint.2018.03.014 29861057

[B53] Raby A. C. Gonzalez-Mateo G. T. Williams A. Topley N. Fraser D. Lopez-Cabrera M.. (2018b). Targeting Toll-like receptors with soluble Toll-like receptor 2 prevents peritoneal dialysis solution-induced fibrosis. Kidney Int. 94, 346–362. doi: 10.1016/j.kint.2018.03.014 29861057

[B54] Raby A. C. Holst B. Le Bouder E. Diaz C. Ferran E. Conraux L.. (2013). Targeting the TLR co-receptor CD14 with TLR2-derived peptides modulates immune responses to pathogens. Sci. Trans. Med. 5, 185ra164. doi: 10.1126/scitranslmed.3005544 23677593

[B55] Raby A. C. Le Bouder E. Colmont C. Davies J. Richards P. Coles B.. (2009). Soluble TLR2 reduces inflammation without compromising bacterial clearance by disrupting TLR2 triggering. J. Immunol. 183, 506–517. doi: 10.4049/jimmunol.0802909 19542461

[B56] Rey Nores J. E. Bensussan A. Vita N. Stelter F. Arias M. A. Jones M.. (1999). Soluble CD14 acts as a negative regulator of human T cell activation and function. Eur. J. Immunol. 29, 265–276. doi: 10.1002/(SICI)1521-4141(199901)29:01<265::AID-IMMU265>3.0.CO;2-G 9933108

[B57] Ridker P. M. Everett B. M. Thuren T. MacFadyen J. G. Chang W. H. Ballantyne C.. (2017). Antiinflammatory therapy with canakinumab for atherosclerotic disease. N Engl. J. Med. 377, 1119–1131. doi: 10.1056/NEJMoa1707914 28845751

[B58] Roh J. S. Sohn D. H. (2018). Damage-associated molecular patterns in inflammatory diseases. Immune Netw. 18, e27. doi: 10.4110/in.2018.18.e27 30181915 PMC6117512

[B59] Roshan M. H. Tambo A. Pace N. P. (2016). The role of TLR2, TLR4, and TLR9 in the pathogenesis of atherosclerosis. Int. J. Inflam 2016, 1532832. doi: 10.1155/2016/1532832 27795867 PMC5067326

[B60] Sanz M. Marco Del Castillo A. Jepsen S. Gonzalez-Juanatey J. R. D’Aiuto F. Bouchard P.. (2020). Periodontitis and cardiovascular diseases: Consensus report. J. Clin. Periodontol 47, 268–288. doi: 10.1111/jcpe.13189 32011025 PMC7027895

[B61] Schiopu A. Cotoi O. S. (2013). S100A8 and S100A9: DAMPs at the crossroads between innate immunity, traditional risk factors, and cardiovascular disease. Mediators Inflammation 2013, 828354. doi: 10.1155/2013/828354 PMC3881579 24453429

[B62] Seaks C. E. Wilcock D. M. (2020). Infectious hypothesis of Alzheimer disease. PloS Pathog. 16, e1008596. doi: 10.1371/journal.ppat.1008596 33180879 PMC7660461

[B63] Shankar M. Uwamahoro N. Backman E. Holmberg S. Niemiec M. J. Roth J.. (2021). Immune resolution dilemma: host antimicrobial factor S100A8/A9 modulates inflammatory collateral tissue damage during disseminated fungal peritonitis. Front. Immunol. 12. doi: 10.3389/fimmu.2021.553911 PMC7953150 33717058

[B64] Sohnle P. G. Hunter M. J. Hahn B. Chazin W. J. (2000). Zinc-reversible antimicrobial activity of recombinant calprotectin (migration inhibitory factor-related proteins 8 and 14). J. Infect. Dis. 182, 1272–1275. doi: 10.1086/315810 10979933

[B65] Tacke F. Alvarez D. Kaplan T. J. Jakubzick C. Spanbroek R. Llodra J.. (2007). Monocyte subsets differentially employ CCR2, CCR5, and CX3CR1 to accumulate within atherosclerotic plaques. J. Clin. Invest. 117, 185–194. doi: 10.1172/JCI28549 17200718 PMC1716202

[B66] Tobias P. S. Curtiss L. K. (2007). Toll-like receptors in atherosclerosis. Biochem. Soc. Trans. 35, 1453–1455. doi: 10.1042/BST0351453 18031244

[B67] Videm V. Albrigtsen M. (2008). Soluble ICAM-1 and VCAM-1 as markers of endothelial activation. Scand. J. Immunol. 67, 523–531. doi: 10.1111/j.1365-3083.2008.02029.x 18363595

[B68] Wirtz T. H. Buendgens L. Weiskirchen R. Loosen S. H. Haehnsen N. Puengel T.. (2020). Association of serum calprotectin concentrations with mortality in critically ill and septic patients. Diagnostics (Basel) 10, 990. doi: 10.3390/diagnostics10110990 33238644 PMC7700375

[B69] Woods J. P. Frelinger J. A. Warrack G. Cannon J. G. (1988). Mouse genetic locus Lps influences susceptibility to Neisseria meningitidis infection. Infect. Immun. 56, 1950–1955. doi: 10.1128/iai.56.8.1950-1955.1988 3397181 PMC259506

[B70] Wu F. Zhang Y. T. Teng F. Li H. H. Guo S. B. (2023). S100a8/a9 contributes to sepsis-induced cardiomyopathy by activating ERK1/2-Drp1-mediated mitochondrial fission and respiratory dysfunction. Int. Immunopharmacol 115, 109716. doi: 10.1016/j.intimp.2023.109716 36652759

[B71] Xu S. Huang Y. Xie Y. Lan T. Le K. Chen J.. (2010). Evaluation of foam cell formation in cultured macrophages: an improved method with Oil Red O staining and DiI-oxLDL uptake. Cytotechnology 62, 473–481. doi: 10.1007/s10616-010-9290-0 21076992 PMC2993859

[B72] Yamanouchi S. Kudo D. Yamada M. Miyagawa N. Furukawa H. Kushimoto S. (2013). Plasma mitochondrial DNA levels in patients with trauma and severe sepsis: time course and the association with clinical status. J. Crit. Care 28, 1027–1031. doi: 10.1016/j.jcrc.2013.05.006 23787023

[B73] Yan L. Bjork P. Butuc R. Gawdzik J. Earley J. Kim G.. (2013). Beneficial effects of quinoline-3-carboxamide (ABR-215757) on atherosclerotic plaque morphology in S100A12 transgenic ApoE null mice. Atherosclerosis 228, 69–79. doi: 10.1016/j.atherosclerosis.2013.02.023 23497784 PMC3640742

[B74] Ye H. Zhou Q. Fan L. Guo Q. Mao H. Huang F.. (2017). The impact of peritoneal dialysis-related peritonitis on mortality in peritoneal dialysis patients. BMC Nephrol. 18, 186. doi: 10.1186/s12882-017-0588-4 28583107 PMC5460447

[B75] Zardawi F. Gul S. Abdulkareem A. Sha A. Yates J. (2020). Association between periodontal disease and atherosclerotic cardiovascular diseases: revisited. Front. Cardiovasc. Med. 7, 625579. doi: 10.3389/fcvm.2020.625579 33521070 PMC7843501

[B76] Zhang Y. Wu F. Teng F. Guo S. Li H. (2023). Deficiency of S100A9 alleviates sepsis-induced acute liver injury through regulating AKT-AMPK-dependent mitochondrial energy metabolism. Int. J. Mol. Sci. 24. doi: 10.3390/ijms24032112 PMC9916677 36768433

