# Supplementary material for: Calprotectin blockade inhibits long-term vascular pathology following peritoneal dialysis-associated bacterial infection
Source: Front Cell Infect Microbiol. 2023 Nov 29;13:1285193. doi: 10.3389/fcimb.2023.1285193 (PMC10716465; doi:10.3389/fcimb.2023.1285193)
Supplement: Supplementary file 1 [file DataSheet_1.pdf]

Supplementary Material

Calprotectin blockade inhibits long-term vascular pathology following peritoneal dialysis-associated bacterial infection

Authors: Esra Cetin, Morgane Mazzarino, Guadalupe T. González-Mateo, Valeria Kopytina, Soma Meran, Donald Fraser, Manuel López-Cabrera, Mario O. Labéta and Anne-Catherine Raby.

Supplementary Table 1. Effect of *S. epidermidis* peritonitis on aortic atherosclerosis-associated gene expression at Day 28

Gene Symbol	Description	Fold Change	*p*
Apoa1	Apolipoprotein A-I	21.7	*0.0001*
Apob	Apolipoprotein B	11.4	*0.0008*
Ccn2	Cellular communication network factor 2	2.3	*0.0001*
Eln	Elastin	2.3	*0.0001*
Fga	Fibrinogen alpha chain	26.3	*0.0001*
Fgb	Fibrinogen beta chain	22.3	*0.0001*
Fn1	Fibronectin 1	2.1	*0.0001*
Hbegf	Heparin-binding EGF-like growth factor	2.3	*0.0002*
Il4	Interleukin 4	2.2	*0.0062*
Itga5	Integrin alpha 5 (fibronectin receptor alpha)	2.1	*0.0001*
Lif	Leukemia inhibitory factor	2.1	*0.0080*
Serpine1	Serine (or cysteine) peptidase inhibitor, clade E, member 1	2.8	*0.0002*
Tgfb2	Transforming growth factor, beta 2	2.5	*0.0011*
Tnc	Tenascin C	2.0	*0.0001*
Vwf	Von Willebrand factor homolog	2.6	*0.0001*
Apoe	Apolipoprotein E	-2.2	*0.0001*
Ccr2	Chemokine (C-C motif) receptor 2	-2.0	*0.0001*
Il5	Interleukin 5	-6.1	*0.0047*
Ppara	Peroxisome proliferator activated receptor alpha	-2.1	*0.0001*

Only statistically significant ($p<0.05$) ≤-2 (in green) or ≥ 2 (in red) fold changes compared to PBS control group are shown.

Supplementary Table 2. Effect of *S. epidermidis* peritonitis combined to PDF exposure, or each separately, on aortic atherosclerosis-associated gene expression at Day 28

Gene Symbol	Description	PDF + *S. epi*		*S. epi*		PDF	
		**Fold Change***	***p***	**Fold Change***	***p***	**Fold Change***	***p***
Abca1	ATP-binding cassette, sub-family A (ABC1), member 1	2.1	*0.0001*	1.8	*0.0029*	3.2	*0.0001*
Apoa1	Apolipoprotein A-I	4.6	*0.0001*	-4.5	*0.0112*	-1.1	*0.5916*
Apoe	Apolipoprotein E	2.1	*0.0002*	2.6	*0.0001*	-1.4	*0.0027*
Bcl2a1a	B-cell leukemia/lymphoma 2 related protein A1a	2.4	*0.0001*	1.8	*0.0010*	1.2	*0.1023*
Bid	BH3 interacting domain death agonist	8.2	*0.0065*	3.1	*0.0001*	7.7	*0.0005*
Birc3	Baculoviral IAP repeat-containing 3	4.0	*0.0002*	1.1	*0.4106*	2.4	*0.0003*
Ccl2	Chemokine (C-C motif) ligand 2	5.9	*0.0235*	5.0	*0.0049*	4.0	*0.0005*
Ccl5	Chemokine (C-C motif) ligand 5	10.7	*0.0001*	-1.1	*0.8549*	3.3	*0.0030*
Ccr2	Chemokine (C-C motif) receptor 2	2.3	*0.0053*	-1.4	*0.1991*	-1.8	*0.3771*
Cdh5	Cadherin 5	2.7	*0.0000*	1.7	*0.0055*	2.2	*0.0033*
Cflar	CASP8 and FADD-like apoptosis regulator	2.1	*0.0002*	1.0	*0.4910*	1.7	*0.0035*
Eng	Endoglin	2.1	*0.0004*	1.3	*0.0034*	2.7	*0.0001*
Fas	Fas (TNF receptor superfamily member 6)	2.2	*0.0006*	1.3	*0.1237*	2.1	*0.0003*
Fga	Fibrinogen alpha chain	4.8	*0.0025*	-3.5	*0.1401*	1.7	*0.3013*
Fgb	Fibrinogen beta chain	6.1	*0.0001*	-3.6	*0.0302*	1.1	*0.7703*
Icam1	Intercellular adhesion molecule 1	2.4	*0.0002*	2.0	*0.0002*	1.6	*0.0089*
Ifng	Interferon gamma	3.5	*0.0098*	-1.2	*0.0325*	-1.4	*0.0060*
Il1a	Interleukin 1 alpha	4.5	*0.0114*	1.9	*0.1577*	5.0	*0.0011*
Il1b	Interleukin 1 beta	9.2	*0.0359*	3.6	*0.1267*	1.7	*0.2044*
Itga2	Integrin alpha 2	3.0	*0.0089*	1.9	*0.1135*	2.6	*0.0033*
Itgax	Integrin alpha X	5.4	*0.0333*	1.8	*0.2391*	2.4	*0.0090*
Itgb2	Integrin beta 2	4.2	*0.0001*	4.6	*0.0003*	2.8	*0.0012*
Kdr	Kinase insert domain protein receptor	2.8	*0.0001*	1.3	*0.0189*	1.3	*0.0566*
Lpl	Lipoprotein lipase	2.3	*0.0001*	2.6	*0.0001*	2.7	*0.0001*
Mmp1a	Matrix metallopeptidase 1a (interstitial collagenase)	2.3	*0.0002*	3.3	*0.0001*	2.5	*0.0001*
Msr1	Macrophage scavenger receptor 1	3.8	*0.0077*	6.9	*0.0001*	3.4	*0.0031*
Nfkb1	Nuclear factor of kappa light polypeptide gene enhancer in B-cells 1	2.1	*0.0002*	1.2	*0.0232*	3.1	*0.0001*
Nr1h3	Nuclear receptor subfamily 1, group H, member 3	3.4	*0.0001*	2.3	*0.0040*	5.1	*0.0001*
Pdgfb	Platelet derived growth factor, B polypeptide	3.7	*0.0001*	1.6	*0.0095*	2.5	*0.0009*
Plin2	Perilipin 2	2.2	*0.0001*	2.3	*0.0001*	2.4	*0.0001*
Ppara	Peroxisome proliferator activated receptor α	2.6	*0.0000*	1.2	*0.0121*	2.9	*0.0000*
Pparg	Peroxisome proliferator activated receptor γ	5.3	*0.0030*	1.6	*0.0175*	5.3	*0.0001*
Rxra	Retinoid X receptor alpha	2.3	*0.0001*	1.4	*0.0359*	4.0	*0.0000*
Sele	Selectin, endothelial cell	2.9	*0.0014*	1.3	*0.2716*	1.1	*0.6213*
Selp	Selectin, platelet	2.1	*0.0190*	1.9	*0.0873*	1.5	*0.1627*
Selplg	Selectin, platelet (p-selectin) ligand	9.5	*0.0002*	2.9	*0.0725*	9.1	*0.0008*
Sod1	Superoxide dismutase 1, soluble	2.5	*0.0001*	2.0	*0.0003*	2.1	*0.0001*
Tnf	Tumor necrosis factor	9.2	*0.0002*	1.8	*0.1647*	-1.4	*0.0060*
Tnfaip3	Tumor necrosis factor, alpha-induced protein 3	8.0	*0.0001*	3.9	*0.0019*	4.8	*0.0162*
Vegfa	Vascular endothelial growth factor A	7.3	*0.0001*	1.5	*0.0019*	5.0	*0.0001*
Ccn2	Cellular communication network factor 2	-2.7	*0.0010*	-2.0	*0.0024*	-1.6	*0.0167*
Fn1	Fibronectin 1	-2.9	*0.0002*	1.3	*0.0024*	-1.5	*0.0007*

Only genes with statistically significant ($p<0.05$) ≤-2 (in green) or ≥ 2 (in red) fold changes for the combination of peritonitis + PDF exposure are shown.
**Compared to PBS control group.*

Supplementary Table 3. Effect of Paquinimod administration on *S. epidermidis* peritonitis-induced changes on atherosclerosis-associated gene expression in aortas at Day28

		S. epi		***S. epi* + Paquinimod**	
**Gene Symbol**	**Description**	**Fold Change***	***p***	**Fold Change***	***p***
Apoa1	Apolipoprotein A-I	21.7	*0.0001*	2.4	*0.0001*
Apob	Apolipoprotein B	11.4	*0.0008*	1.6	*0.0635*
Ccn2	Cellular communication network factor 2	2.3	*0.0001*	1.2	*0.0005*
Eln	Elastin	2.3	*0.0001*	1.6	*0.0002*
Fga	Fibrinogen alpha chain	26.3	*0.0001*	2.2	*0.0006*
Fgb	Fibrinogen beta chain	22.3	*0.0001*	1.3	*0.2417*
Fn1	Fibronectin 1	2.1	*0.0001*	1.6	*0.0001*
Hbegf	Heparin-binding EGF-like growth factor	2.3	*0.0002*	-1.3	*0.9125*
Il4	Interleukin 4	2.2	*0.0062*	1.6	*0.2511*
Itga5	Integrin alpha 5 (fibronectin receptor alpha)	2.1	*0.0001*	1.5	*0.0003*
Lif	Leukemia inhibitory factor	2.1	*0.0080*	-1.8	*0.0130*
Serpine1	Serine (or cysteine) peptidase inhibitor, clade E, 1	2.8	*0.0002*	1.1	*0.0816*
Tgfb2	Transforming growth factor, beta 2	2.5	*0.0011*	1.1	*0.0118*
Tnc	Tenascin C	2.0	*0.0001*	1.2	*0.0184*
Vwf	Von Willebrand factor homolog	2.6	*0.0001*	-1.4	*0.0013*
Apoe	Apolipoprotein E	-2.2	*0.0001*	-1.2	*0.0022*
Ccr2	Chemokine (C-C motif) receptor 2	-2.0	*0.0001*	-1.1	*0.3971*
Il5	Interleukin 5	-6.1	*0.0047*	-3.3	*0.0147*
Ppara	Peroxisome proliferator activated receptor alpha	-2.1	*0.0001*	1.0	*0.3172*

Only genes with statistically significant ($p<0.05$) ≤ -2 (in green) or ≥ 2 (in red) fold changes following S. epidermidis administration are shown.
** Compared to PBS control group.*

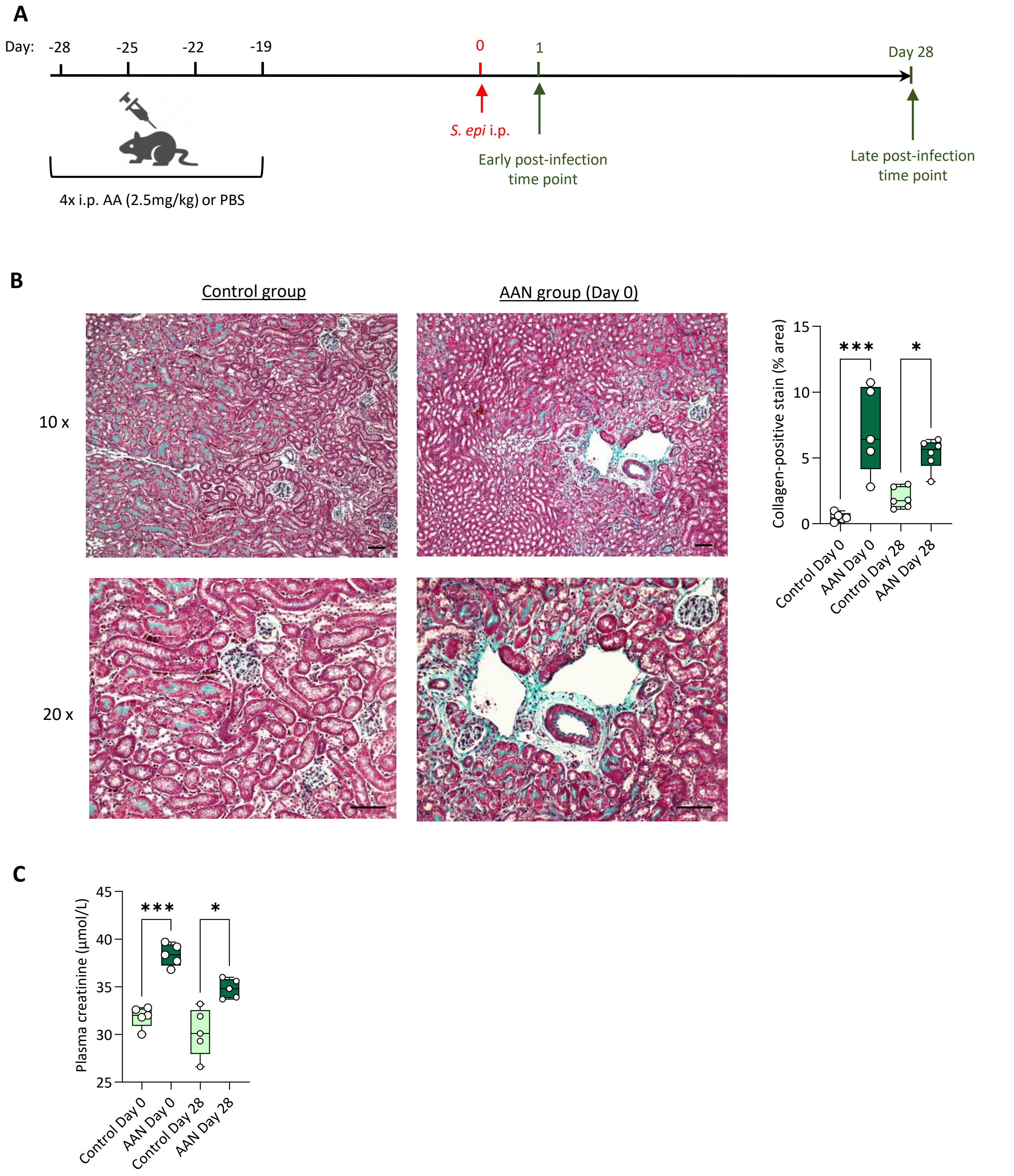

Supplementary Figure 1. Kidney damage and loss of function following repeated AA injections in mice
A-C C57BL/6J mice (n=5 /group) were injected intraperitoneally with AA (2.5mg/kg) or PBS on days -28, -25, -22 and -19 to induce chronic nephropathy. Day 0 is the day of *S. epidermidis* administration. AAN was verified at Day 0 and Day 28 by the development of kidney fibrosis (**B**) and a significant elevation in plasma creatinine (**C**). **B**. Representative images of Masson trichrome stain of kidneys from a healthy mouse (left panels) and a mouse with chronic AAN (right) . Cytoplasm is stained red, nuclei are in dark brown, and collagen is stained blue, identifying renal fibrosis. Scale bars: 100 µm. Graph shows the percentage of collagen positive stain for each group (3 non-overlapping fields of view scored for each of 5 animals/group) **C**. Creatinine measurements in plasma. *, p<0.05; ***, p<0.005, ordinary one-way ANOVA (normal distribution) or Kruskal-Wallis test (non-normal distribution).

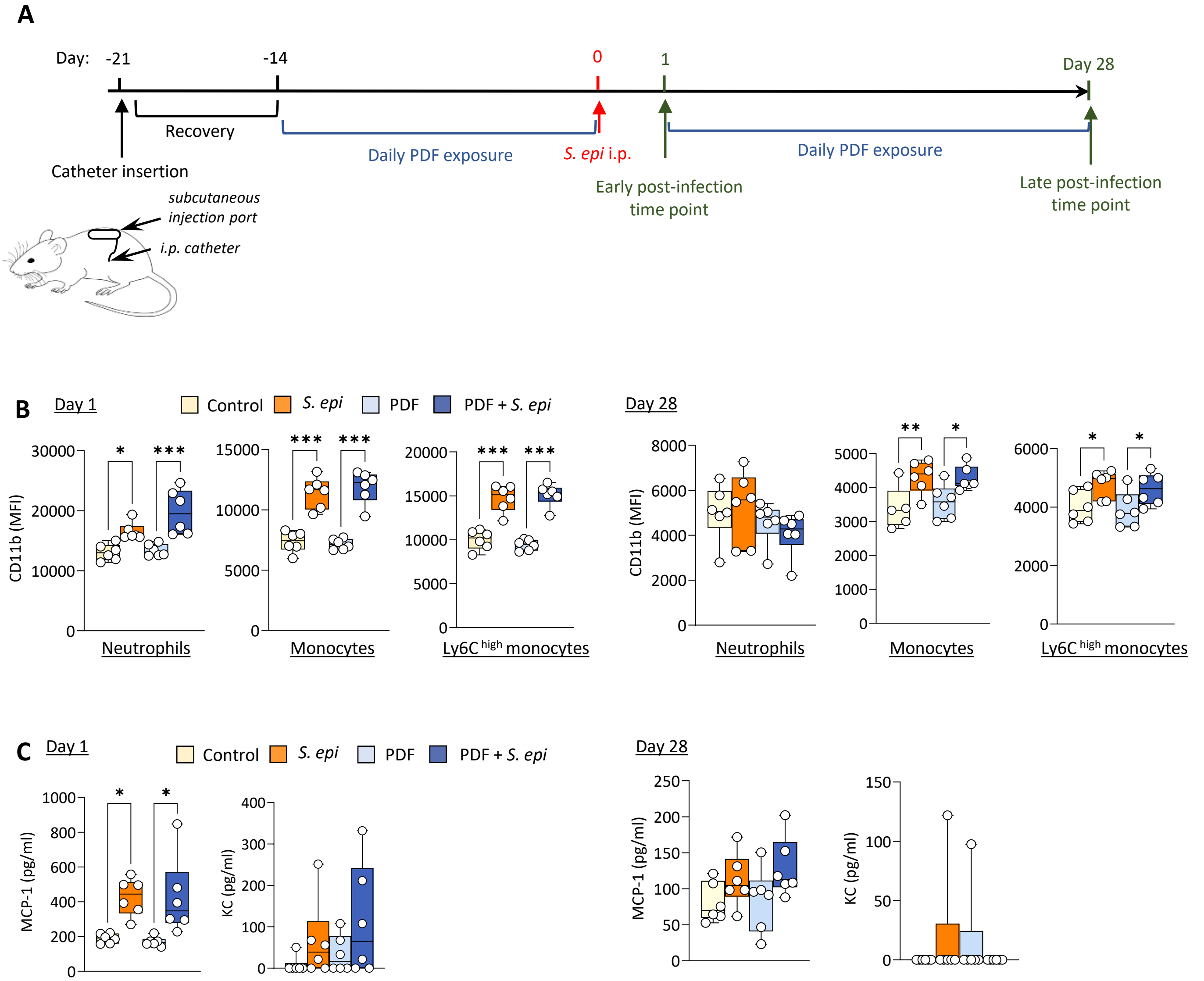

Supplementary Figure 2. Routine PDF exposure in mice does not affect *S. epidermidis*-induced changes in blood innate leukocyte CD11b expression and cytokine levels.

A-C C57BL/6 mice (n=6/ group) were fitted with a peritoneal catheter, given a 7-day recovery period and instilled once daily with 2ml PBS or PDF for 14 day. Mice were then i.p. injected with *S. epidermidis* or PBS (Day 0) and culled at Day 1 or further exposed daily to PBS or PDF, prior to culling at Day 28 (**A**). Blood was collected and expression of CD11b on innate immune leukocytes was determined by flow cytometry (**B**) and plasma levels of cytokines were determined by ELISA (**C**). *, p<0.05; **, p<0.01; ***, p<0.005, ordinary one-way ANOVA (normal distribution) or Kruskal-Wallis test (non-normal distribution).

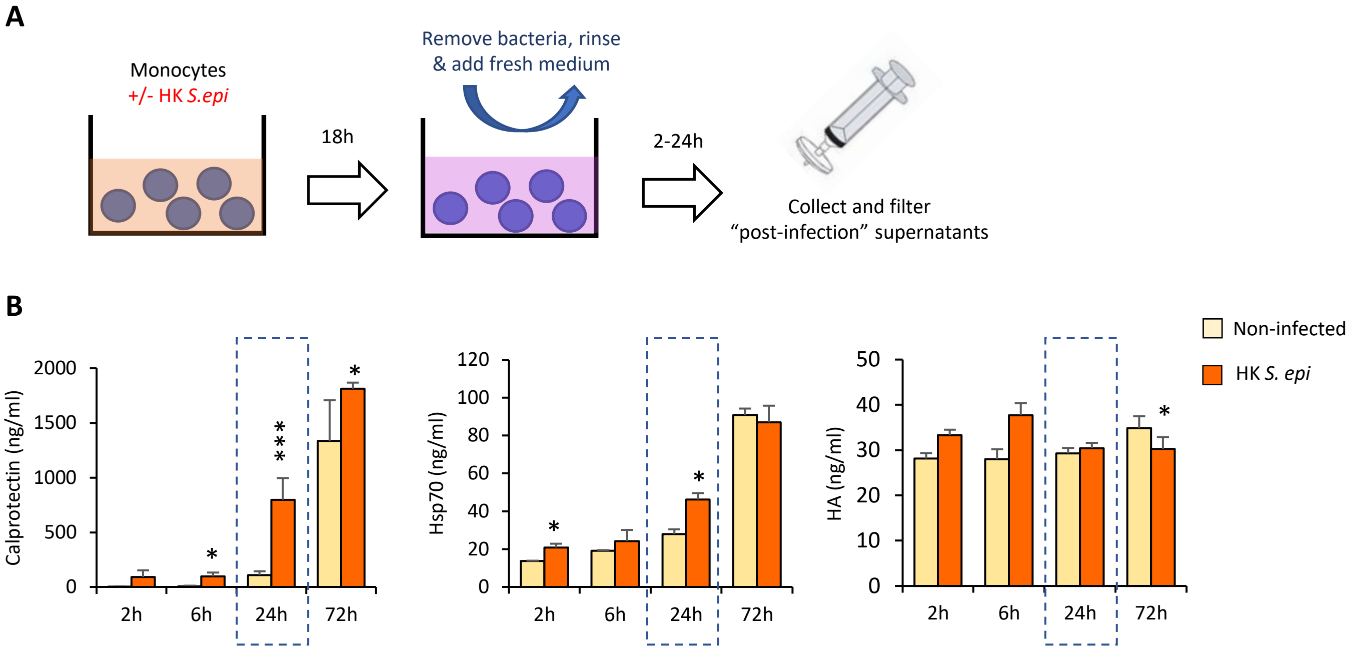

Supplementary Figure 3. Preparation of Calprotectin-containing post-infection supernatants

Mono-Mac 6 monocytes were stimulated (18h) with heat-killed *S. epidermidis* (HK *S. epi*, 5x10[8]cfu/ml) or left untreated. Supernatants were removed, cells were washed and further cultured in complete medium for the indicated durations before post-infection supernatant collection and filtration (0.22µm) (**A**). Calprotectin, Hsp70 and HA levels were determined by ELISA (**B**). 24h post-infection supernatants were selected for use in functional experiments, as the increase in Calprotectin and Hsp70 compared to non-infected supernatants was highest at this time point. *, $p<0.05$; ***, $p<0.005$; HK *S. epi* vs Non-infected, unpaired Student t-test.
